# Targeting CD276 with Adapter-CAR T-cells provides a novel therapeutic strategy in small cell lung cancer and prevents CD276-dependent fratricide

**DOI:** 10.1186/s13045-025-01729-8

**Published:** 2025-07-28

**Authors:** Beate Kristmann, Niels Werchau, Lakshmi Suresh, Elisabeth L. Pezzuto, Sophia Scheuermann, Simon Krost, Karin Schilbach, Moustafa Moustafa-Oglou, Anna-Sophia Mast, Miriam Droste, André Felsberger, Lukas Kiefer, Pierre Abramowski, Lars Zender, Joerg Mittelstaet, Christian M. Seitz

**Affiliations:** 1 Present Address: Department of General Pediatrics, Hematology and Oncology, University Children’s Hospital Tuebingen, Tübingen, Germany; 2 Present Address: Institute for Medical Genetics and Applied Genomics, University Hospital Tuebingen, Tübingen, Germany; 3 R&D Department, Miltenyi Biotec B.V. & CO. KG, Bergisch Gladbach, Germany; 4 Present Address: Department of Internal Medicine III, University Hospital Regensburg, Regensburg, Germany; 5 Present Address: University of Tuebingen, iFIT Cluster of ExCellence (EXC2180) “Image-Guided and Functionally Instructed Tumor Therapies”, Tuebingen, Germany; 6 Present Address: German Cancer Research Consortium (DKTK), Partner Site Tuebingen, German Cancer Research Center (DKFZ), Heidelberg, Germany; 7 Present Address: Faculty of Biosciences, Heidelberg, Germany; 8 Present Address: Hopp Children’s Cancer Center Heidelberg (KiTZ), Heidelberg, Germany; 9 Present Address: Department of Pediatric Oncology, Hematology, and Immunology, Heidelberg University Hospital, Heidelberg, Germany; 10 Present Address: B310 Clinical Cooperation Unit Pediatric Oncology, German Cancer Research Center (DKFZ), Heidelberg, Germany; 11 Department of Medical Oncology & Pulmonology (Internal Medicine VIII), University Hospital Tuebingen, Tübingen, Germany; 12 Present Address: Faculty of Life Sciences, Reutlingen University, Reutlingen, Germany

**Keywords:** SCLC, CD276, Immunotherapy, AdCAR-T, Fab-based adapter molecule, Fratricide, In vivo solid tumor targeting

## Abstract

**Background:**

Survival rates in Small Cell Lung Cancer (SCLC) remain dismal, posing a huge medical need for novel therapies. T-cells, engineered to express chimeric antigen receptors (CAR-T) have demonstrated clinical activity against a variety of haematological malignancies. Yet, efficacy against solid tumour entities remains limited.

**Methods:**

In this study, we investigated the expression of CD276 (B7-H3), an immune checkpoint molecule and promising target antigen for CAR-T therapy in SCLC, at the RNA and protein level. We further developed novel Fab-based adapter molecules (AM) targeting CD276 and optimized our previously established modular Adapter CAR-T (AdCAR-T) platform as well as AM dosing schemes.

**Results:**

CD276 is broadly expressed across SCLC subtypes, representing a promising target for CAR-T therapy. We describe that T-cell activation and CAR-signalling induces CD276-expression on CAR-T, resulting in CD276-dependent fratricide, limiting anti-CD276-CAR-T expansion and activity. The AdCAR-T platform allows CAR-T expansion in absence of CD276 targeting. Novel CD276 targeted AMs demonstrate potent in vitro and in vivo activity against SCLC. Intermittent AM-dosing allows functional persistence of AdCAR-T in vivo in contrast to CD276-targeted conventional CAR-T. AdCAR-T in vivo expansion and activity is further promoted by introducing activation-induced, AM remote controlled, IL-18 secretion into the AdCAR-T design.

**Conclusion:**

We identified CD276 as a promising target antigen, uniformly expressed in SCLC and demonstrate the therapeutic potential of novel anti-CD276 Fab-based AM in combination with optimized, IL-18 armoured AdCAR-T.

**Supplementary Information:**

The online version contains supplementary material available at 10.1186/s13045-025-01729-8.

## Background

Small cell lung cancer (SCLC) accounts for over 15% of lung cancer cases and is marked by aggressive behaviour and poor survival rates, with current treatments having limited success. In contrast to advancements in non-small cell lung cancer (NSCLC), SCLC survival rates have stagnated, highlighting the urgent need for novel therapies. Chimeric antigen receptor T-cell (CAR-T) therapy has successfully shown effectiveness in haematological cancers in various studies, but its application to solid tumors like SCLC faces significant challenges such as T-cell exhaustion and limited CAR-T efficacy. In this study, we address these challenges by targeting the transmembrane protein CD276 with the previously described AdCAR-T system. We aim to demonstrate the in vitro and in vivo efficacy of AdCAR-T with our novel Fab-based adapter molecule, hereby addressing the challenges of conventional CAR-T as well as immunotherapies against solid tumors.

## Introduction

Small cell lung cancer (SCLC) remains a challenging disease with limited treatment options, accounting for over 15% of lung cancer cases worldwide [1, 2]. Its aggressive nature is characterized by short doubling times and high metastasis rates which result in poor outcomes for the patients [3, 4]. Survival rates for non-small cell lung cancer (NSCLC) significantly improved due to the advent of innovative treatment options, especially immune checkpoint blockade (ICB) [5]. In contrast, SCLC survival rates have stagnated over the past three decades [6]. While the current standard treatment of radiotherapy and platinum-based chemotherapy often leads to remission, most of the patients experience early and systemic relapses with a median survival of seven months, [2, 6, 7]. The high relapse rate highlights the need for novel therapeutic approaches to address minimal residual disease. Genomically, SCLC is often characterized by the inactivation or alterations of the tumor suppressor genes *TP53* and *RB1* [8]. In contrast, genetically the disease is rather a heterogenous one. Rudin et al. recently defined four different subtypes that are used to reassess individual therapeutic approaches [9]. The classification is based on the differential expression of four transcription regulators: *ASCL-1*, *NEUROD1*, *POU2F3* and *YAP1*. Based on RNA-expression of those markers, the subclassifications of SCLC-A, SCLC-N, SCLC-P and SCLC-Y was introduced and could be used to identify therapeutic weaknesses and challenges [10].

In recent decades, chimeric antigen receptor T-cells (CAR-T) have emerged as an effective therapy, particularly in haematological malignancies [11, 12]. FDA-approved CAR-T therapies have achieved high remission rates in B-cell acute lymphocytic leukaemia (B-ALL), Non-Hodgkin lymphoma (NHL), and multiple myeloma (MM) and these therapies have led to improved overall survival rates for patients [13, 14]. However, this therapeutic efficacy remains to be mimicked against solid tumor entities [15]. Many factors contribute to therapeutic failure of CAR-T, such as a hostile tumor microenvironment (TME) promoting exhausted and dysfunctional T-cells, or tumor escape through selection of antigen-low or -negative subclones in heterogeneous diseases [16–18]. To address these and other limitations of CAR-T therapy, we developed the modular Adapter-CAR-T-cell (AdCAR-T) platform [19–21]. AdCAR-Ts are directed against a biotin-tag, referred to as a linker-label epitope (LLE), that can be conjugated to various types of binding molecules, e.g., monoclonal antibodies (mAbs) or fragments thereof, referred to as adapter molecule (AM). AMs mediate AdCAR-T binding to surface antigens and subsequent CAR-signalling and T-cell activation. We have previously demonstrated that AdCAR-Ts allow for precise control over effector cell function, can be redirected against a variety of different antigens, and allows simultaneous and sequential targeting of multiple antigens, paving the way for personalised combinatorial targeting approaches [19–21].

CD276, also known as B7-H3, is a type I transmembrane protein of the B7-costimulatory family [22]. CD276 is highly expressed on tumor cells and tumor vasculature in a variety of solid tumor entities, while it is expressed at a low levels on healthy tissue [23]. Therefore, CD276 is a promising CAR-T target that has already been evaluated both preclinically and in early clinical trials in solid peripheral tumors and CNS malignancies [24–27]. The relevance of CD276 in SCLC remains to be explored. In the present study, we investigated CD276-expression in SCLC and evaluated CAR-T and AdCAR-T-based targeting of CD276 as a novel and highly promising therapeutic strategy against SCLC.

## Material & methods

### DLL3 and CD276 expression on primary patient samples and SCLC cell lines

The gene expression of CD276 and DLL3 was analysed using the dataset ´ps_avgpres_gse60052counts82_tpm109geo´ [28]. The mRNA expression of DLL3 and CD276 was obtained from a dataset provided by Tlemsani et al. (2020). The tool can be accessed here: https://discover.nci.nih.gov/rsconnect/SclcCellMinerCDB/.

### Analysis of CD276-expression on primary patient samples

SCLC primary tumor samples (*n* = 46) and reference tissue were obtained from the biobank of the University Hospital Tuebingen as FFPE blocks. Informed consent was obtained from all patients or their guardians for use of their samples for research, as approved by the ethics committee of the University Hospital Tuebingen in accordance with the Declaration of Helsinki (ethics approval No. 508-2016BO1). In collaboration with the Institute of Pathology and Neuropathology Tuebingen, we generated a Tissue microarray (TMA) by defining cores of the tumor regions with a diameter of 2 mm and assembling the TMA using the TMA Grand Master (3DHISTECH Kft.). CD276 expression was analysed by classical IHC staining (Vector Laboratories, #LS-C743430-3) by the Institute of Pathology and Neuropathology Tuebingen. Liver and Tonsil were used as reference tissue. From the scanned slides, the cells of a tumor region were segmented using QuPath and CD276-positive cells were annotated. For the tumor region, we performed an expression intensity scoring on a four-point scale: 0 no expression, 1 low expression, 2 medium expression, and 3 high expression. H-Score was then calculated by multiplying the score with the percentage of positive tumor cells in the tumor section.

### CD276-binder generation and high-throughput functional screening

To generate human CD276-specific antibody fragments, two fully human naïve scFv phage display libraries (κ and λ isotypes; constructed at Miltenyi Biotec) were employed. For selection, recombinant CD276 protein was immobilized on high-binding 96-well ELISA plates and blocked to prevent nonspecific interactions. Following a pre-clearing step in blocked wells, the libraries were transferred to the antigen-coated wells. After completing three rounds of panning, individual clones were analysed for CD276 binding via flow cytometry. Affinity maturation was applied to selected parental clones to enhance binding characteristics and sequence diversity. This was achieved using error-prone PCR and light chain shuffling, resulting in a set of scFv variants. From these, unique antibody clones were identified after computational exclusion of sequences containing predicted N-glycosylation sites or unpaired cysteine residues. The resulting scFv panel was transiently expressed and screened for specific binding to CD276-positive OCI-AML-2 cells in contrast to CD276-negative SUP-T1 cells using flow cytometry. From this set, candidates demonstrating selective target recognition were selected for further functional assessment. Functional activity was evaluated via an automated high-throughput cytotoxicity assay performed over three consecutive rounds of co-culture. OCI-AML-2 cells were used as targets, while effector cells consisted of sAdCAR-Ts directed against a recombinant tag fused to the Fab. Adapter molecules were added at a final concentration of 50 ng/mL during each round. The effector-to-target (E: T) ratio was maintained at 2.5:1, and tumor cell lysis was quantified after 96 h by flow cytometry. Selected scFv fragments were transiently produced as tag free Fab fragments in mammalian expression systems and purified using the CaptureSelect IgG-CH1 Affinity Matrix (Thermo Fisher Scientific). Protein concentrations were measured by absorbance at 280 nm. Thermal stability was assessed using the Tycho system (NanoTemper Technologies), with inflection temperatures determined from fluorescence shifts during a thermal ramp, as per the manufacturer’s protocol. Quality control included SDS-PAGE and LC-MS analysis of intact Fab mass. For site-specific labelling, Fabs were biotinylated using LC-LC-biotin. Successful conjugation was verified by LC-MS. Biotinylated Fabs were subsequently used in flow cytometry-based binding assays, wherein 1 × 10⁵ CD276-positive OCI-AML-2 cells were sequentially stained with 1 µM biotinylated Fab (1 h, 4 °C), followed by a staining with anti-biotin antibody (Miltenyi Biotec; 15 min, 4 °C), and were analysed via flow cytometry.

### Flow cytometry analysis

Dead cells were excluded with 7AAD (BD). FACS staining was performed in a total volume of 100 µL CliniMACs (Miltenyi Biotec) for 15 min in the dark (4 °C). After washing (3 mL CliniMACs) cells were analysed in 200 µL CliniMACs with a BD FACSCanto™ II flow cytometer (BD Biosciences). Data were analysed using the FlowJo software (v10). Full antibody information is provided in Additional Tables 1 and Table 2.

### Cell culture and cell lines

DMS114 (Cat# CRL-2066), SJCRH30 (Cat# CRL-2061), Raji (Cat# CCL-86), Sup-T1 (Cat# CRL-1942), NCI-H446 (Cat# HTB-171) and IMR-32 (Cat# CCL-127) were purchased from the American Type Culture Collection (ATCC); SHP-77-Luc-GFP; NCI-H196 were provided by D. Sonanini (WSIC, Tuebingen, Germany); OCI-AML-2 (Cat# ACC gg) were purchased from the Deutsche Sammlung von Mikroorganismen und Zellkulturen (DSMZ) GmbH (Braunschweig, Germany). Cells were cultured in RPMI (Sigma Aldrich) supplemented with 2 mM-Glutamine, 100 U/mL penicillin, 100 µg/mL streptomycin, and 10% heat inactivated FBS (Thermo Fisher Scientific). OCI-AML-2 cells were cultured in alpha-MEM (Sigma Aldrich) supplemented with 20% FBS. All cells were cultured at 37 °C containing 5% CO_2_. For in vivo imaging and in vitro bioluminescence based cytotoxicity assays, tumor cell lines were transduced using a lentiviral vector (LV) encoding a firefly luciferase (eFFly; Plasmid #104834, Addgene) and sorted for GFP-expression by flow cytometry to achieve high purity of eFFly-expressing cells.

### Human T-cell isolation and cultivation

Lymphocytes were isolated from human donor blood (approved by the Institutional Ethical Review Board 761/2015BO2) via gradient centrifugation using BioColl^®^ (Bio&Sell). CD4/CD8-positive T-cell isolation was performed with the MACS Cell Separation kit (Miltenyi Biotec) following the manufacturer’s protocol. T-cells were first activated using TransAct (Miltenyi Biotec) and further cultured in TexMACS media (Miltenyi Biotec) supplemented with 10 ng/mL IL-7, 5 ng/mL IL-15, 100 U/mL penicillin, and 100 µg/mL streptomycin. The cell concentration was kept between 0.5 and 1 × 10^6^ cells/mL during cultivation. For the analysis of CD276-expression the T-cells were activated with either TransAct, OKT3 (Biotec, CD3-Antibody) or only with the cytokines IL-7 and IL-15. T-cell subtypes were analysed with CD45RA (APC; Miltenyi Biotec) and CD62L (FITC, Miltenyi Biotec) marker expression.

### Lentiviral vector construction and transduction of human T-cells

All constructs include a signal peptide, a target specific scFv, an extracellular spacer or hinge domain and a CD8 transmembrane domain. As third generation CAR-constructs, they additionally contained a 4-1BB/CD28 co-stimulatory, as well as CD3ζ. The CD19-CAR encodes the sequence of FMC63-derived scFv [29]. For the AdCAR_IL-18 construct human IL-18 encoding sequence was placed after a 3´NFAT-inducible NFATsyn promotor containing six consensus NFAT enhancer repeats and a synthetic TATA box, followed by a PGK promotor driving constitutive AdCAR expression. LV particles were generated by transient transfection of HEK 293 T-cells using polyethyleneimine. LV-containing supernatants were concentrated by overnight centrifugation (5380 x*g*, 24 h) and pelleted LVs were resuspended in PBS before storage at − 80 °C. LV-titres were determined by the transduction of SupT1 and flow cytometry analysis for ∆LNGFR expression. Human primary T-cells were activated with TransAct (Miltenyi Biotec) according to the manufacturer’s protocol. After 36 h, 2 × 10^6^ cells were transduced in a volume of 200 µL media at a MOI of 10 with the LVs encoding for different third generation CAR constructs (AdCAR, AdCAR encoding IL-18 (AdCAR_IL-18), directly CD19-targeting CAR (CD19-CAR) and directly CD276-targeting CAR (dCAR). After 90 min of spinoculation (600 x*g*, 34 °C), the transduced cells were cultivated in the initial 200 µL media for 4 h 30 min before adding 1800 µL media. After nine days of culture, CAR expression was analysed by flow cytometry by staining first with 7AAD (BD Biosciences) to identify living cells, second with AdCAR Detection reagent (PE, Miltenyi Biotec). For CAR-expression detection in dCAR-T, the cells were incubated for 30 min at 4 °C with 1 µg/mL CD276-pProtein in 100 µL PEB (Thermo Fisher; **#** RP-87983). After a washing step with 120 µL PEB cells were stained with HIS-antibody (PE, Miltenyi Biotec) according to the manufacturer’s protocol and analysed in 200 µL PEB (Additional file 1).

### Proliferation assay

In a 6-well G-Rex6M Multi-Well Cell Culture Plate, 5 × 10^6^ T-cells (either un-transduced (UTD), dCAR-expressing, or AdCAR-expressing) were seeded in TexMACS media (Miltenyi Biotec). Every other day, the cells received fresh cytokines (12.5 ng/mL IL-7/IL-15). Cells were quantified on day six of cell culture via flow cytometry. On the days of analysis, CD276-expression was assessed by flow cytometry analysis in addition to the cell number (CD276-PE antibody, Miltenyi Biotec).

### Cytotoxicity assay

AdCAR-T were co-incubated with 2 × 10^5^ luciferase-transduced tumor cells (IMR-32, DMS114, RH30, SHP-77) at an E: T ratio of 8:1, 4:1, 2:1, 1:1, 1:2, 1:4 and 1:8, in the presence of 5 nM AM in 200 µL RPMI. To detect the tumor cells’ luciferase activity, the substrate D-luciferin was added (4 µg/mL). Specific target cell lysis was calculated via bioluminescence signal detection using the SPARK^®^ microplate reader (Tecan Group) after 24 h, 48 h, and 72 h incubation time respectively. The effect size of the specific tumor cell lysis was calculated by first normalizing the treatment groups of AdCAR-T + AM-candidates to the tumor cell only group at each time point respectively. From the resulting viability of the tumor cells, the specific lysis was calculated (1-% of viability).

### Co-Culture assay

CD19-CAR-T or activated T-cells (Mock) were co-cultured with Raji at an E: T of 1:2 in a T75 cell culture flask (Greiner Bio-One) in 12 mL RPMI. CAR-T were re-challenged with 4 × 10^6^ CD19-expressing Raji cells every 24 h. After 72 h, final analysis was performed with life /dead staining (7AAD, BD), combined with markers specific for target cells (CD20-PE, Miltenyi Biotec), CAR-T-cells (CD19-Biotin and Biotin-PE, Miltenyi Biotec), as well as CD276-expression (CD276-BUV737, BD).

### AdCAR-T fratricide assay

Ten-day old AdCAR-T, dCAR-T, or activated T-cells were seeded in a 6-well plate (Greiner Bio) in 3 mL TexMACS medium (Miltenyi Biotec) at a density of 0.5 × 10^6^ cells/mL. Either AM46 (10 ng/mL), a biotinylated IgG1 aCD276-mAb (10 ng/mL, Miltenyi Biotec), or no antibodys were added. Cells were stained for dead cells (7AAD, BD), activation markers (CD69-FITC, CD25-PE-Vio-770, Miltenyi Biotec) and CD276-expression (APC, Miltenyi Biotec) every 24 h for 72 h in total.

### Re-challenge assay

2 × 10^5^ luciferase-expressing tumor cells (IMR-32, DMS114, RH30, SHP-77) were seeded in a 96-well plate and co-incubated with AdCAR-T at an E: T of 4:1 and an AM concentration of 5 nM in 200 µL RPMI. Luciferin was added at a concentration of 4 µg/mL. Every 24 h, specific bioluminescence was analysed with the SPARK^®^ microplate reader (Tecan Group) and specific target lysis calculated. Every 48 h the AdCAR-T were transferred to a new plate pre-seeded with target cells, and re-challenged with the same E: T ratio. AMs and D-Luciferin were re-supplemented each cycle to match starting conditions. The effect size of the specific tumor cell lysis was calculated by first normalizing the treatment groups of AdCAR-T + AM-candidates to the tumor cell only group at each time point respectively. From the resulting viability of the tumor cells, the specific lysis was calculated (1-% of viability).

### Cytokine analysis

Cytokines in cell supernatants were determined with the MACSPlex Assay (Miltenyi Biotec Biotec) following the manufacturer’s protocol.

### EC_50_ assay

To determine the EC_50_ of the AdCAR system, AdCAR-T were seeded with 2 × 10^5^ RH30 at an E: T ratio of 2:1 in 200 µL RPMI in a 96-well plate. The AM concentration was diluted stepwise from 0.3 nM to 0.3 pM in three 10-fold dilution steps. To each well, D-Luciferin (4 µg/mL) was added and after 24 h, 48 h, and 72 h the bioluminescence of the tumor cells was detected with the SPARK^®^ microplate reader (Tecan Group). From this, the target cell lysis was calculated. The EC50 was then calculated with Graph Pad Prims (v10) by performing a non-linear regression.

### Xenogeneic mouse model

For the in vivo evaluation of the AdCAR system in SCLC, female NOD.Cg-*Prkdc*^*scid*^ *Il2rg*^*tm1WjI*^/SzJ (6 weeks old, Order #614NSG) were purchased from Charles River (Sulzfeld, Germany). At 8 weeks of age, the mice were used for the experiments. 0.5 × 10^6^ SHP-77 were injected into the lateral tail vein (*i.v.*) of the mice. Three days later, 10 × 10^6^ or 30 × 10^6^ AdCAR-T were injected *i.v*. and the mice received the AM (100 µg) either daily, every other day, or for four days followed by three days break. Control groups without AM received PBS. Tumor growth was monitored through bioluminescence imaging (BLI) with the IVIS Lumina (Perkin Elmer). For the respective groups, mice were re-challenged with 0.5-1 × 10^6^ SHP-77 *i.v.*, respectively, at day 46 or with 1 × 10^6^ SHP-77 at day 38. As soon as the mice reached the predetermined health score, evaluated by the experimenter, the mice were excluded from the experiment. All studies were carried out in accordance with the guidelines of the Federation of European Laboratory Animal Science Associations (FELASA) in the animal facilities of the University Children´s Hospital Tuebingen, Germany. Experiments were conducted according to the standards of the ethics committee board and animal care committee of the regional administrative council of Tuebingen (approval number K04/21G). The maximum tumor burden was not exceeded (Ullman-Cullere score < 3). Whole mouse blood was first cleared from erythrocytes with erythrocyte lysis buffer (c.c. Pro Gmbh) following the manufacturer’s protocol. The remaining cells were stained with the following Antibodies: CD45, murine CD45, CD4, CD8, CD276, CD223, CD366, CD279, CD62L, CD45RA (full antibody information provided in Additional Table 1).

### Statistical analysis and quantification

For the comparison between two groups, the Wilcoxon Test or a t-test were used. For comparisons between multiple groups with several variances, two-way ANOVA was used to analyse statistical significance. A *p*-value < 0.05 indicates statistical significance. Sample numbers (n) indicates number of biological replicates except when stated otherwise in the figure legends. Statistical analysis was performed on all results. Statistical significance is indicated with brackets and asterisks, if absent the differences were not significant. All statistical analysis was performed using Graph Pad Prism (v10) software.

## Results

### CD276 is highly expressed on SCLC across different subtypes

**Fig. 1:**
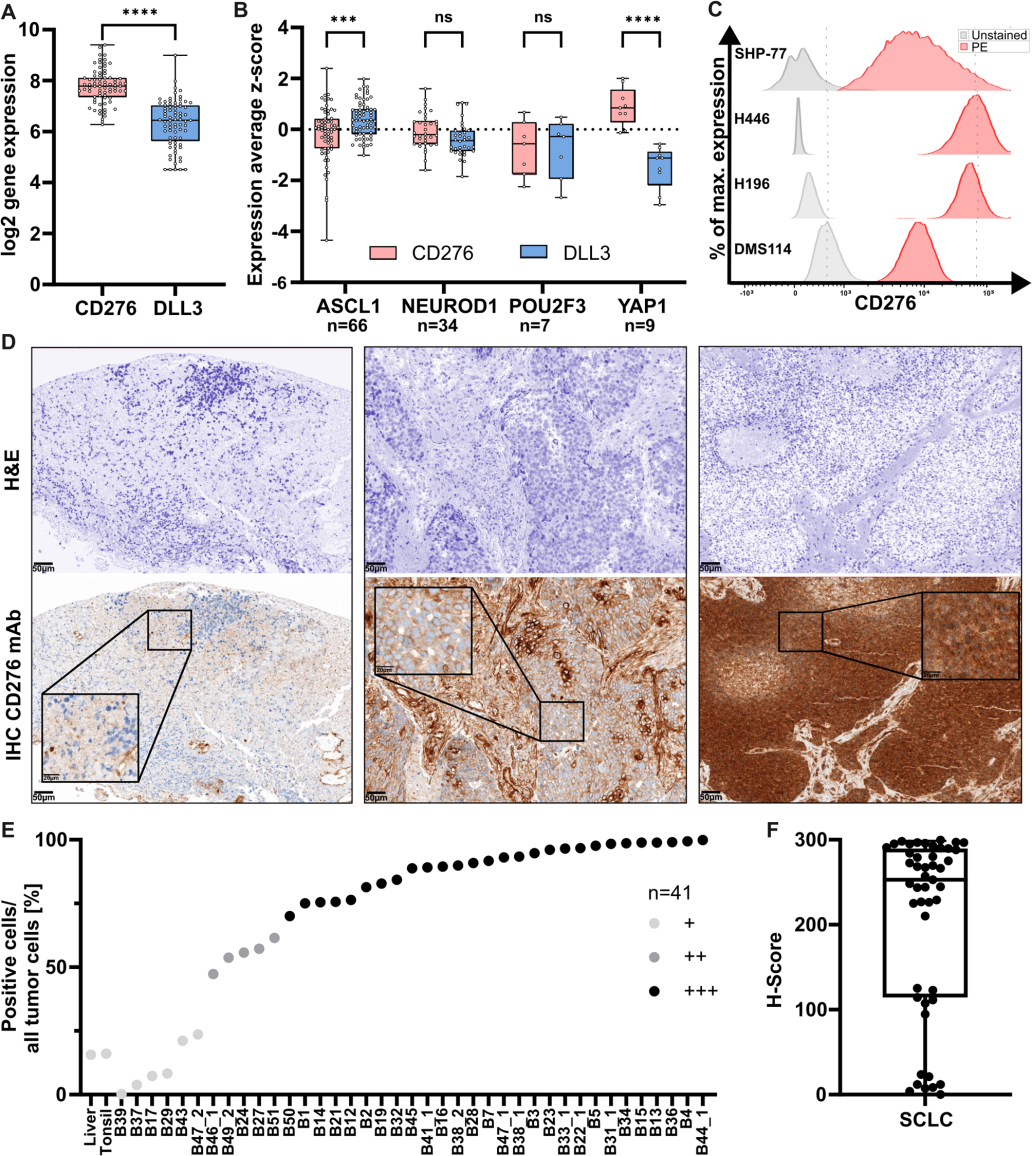
CD276-expression is high on SCLC primary patient samples. (**A**) Analysis of 79 primary patient samples showed a higher gene expression of CD276 compared to DLL3. *n* = 79. ****p < 0.0001 (unpaired t-test). (**B**) Based on the mRNA analysis of cell lines derived from tumor patient samples, CD276 is broadly expressed on all four subtypes of SCLC. CD276-expression is significantly higher than DLL3-expression in the subtypes ASCL1 and YAP1. *n* as shown. ***p < 0.001. ****p < 0.0001 (Two-way ANOVA). (**C**) CD276-expression on four different SCLC cell lines was analysed via flow cytometry and showed high expression on all. (**D**) IHC staining of CD276 on a TMA of 39 primary patient samples was evaluated. Exemplary pictures show low expression (left; score 1), medium expression (middle; score 2) and high expression (right; score 3). Scale bar represents 50 μm. Positive cell to negative cell ratio was calculated from tumor regions (exemplarily highlighted by square) and showed (**E**) high expression in 71.8% of the samples. (**F**) H-score was calculated by multiplying the percentage of positive cells with the score

To understand the clinical relevance of CD276 in SCLC, we determined expression levels of CD276 on SCLC in different primary patient sample cohorts and tumor cell lines. Using an RNA sequencing dataset of 79 primary SCLC patient samples by Tlemsani et al., we analysed the transcript expression of CD276 [30]. At RNA level, CD276-expression was found to be significantly higher in contrast to clinically evaluated target antigens such as DLL3 (Fig. 1A) [31]. The z-score of cell lines, subclassified into the four different known subtypes of SCLC (SCLC-A, SCLC-N, SCLC-P, SCLC-Y) revealed homogeneous expression of CD276 across all subtypes (Fig. 1B). DLL3 was found to be heterogeneously expressed in the subtypes, with low expression levels in the SCLC-Y subtype and CD276 significantly higher expressed compared to DLL3 in both the SCLC-A (ASCL1) and SCLC-Y (YAP1) subtypes (Fig. 1B). To confirm surface protein expression of CD276, a panel of SCLC cell lines, SHP-77 (SCLC-A), H446 (SCLC-N), H196 (SCLC-Y), and DMS114 (SCLC-P), was analysed by flow cytometry, verifying high CD276-expression at protein level compared to Isotype control (Fig. 1C). Lastly, we confirmed CD276-expression by IHC on a TMA containing 39 primary patient tumor samples. Tumor regions, as indicated by squares, were analysed for their percentage of CD276-positive cells (Fig. 1D). 71.8% of primary patient tumor samples express CD276 at the highest level (+++, Fig. 1E). Applying a scoring scheme from 0 (no expression) to 3 (high expression), the calculated H-score depicted high staining intensity for 28 out of 39 patients (Fig. 1F). Our data clearly demonstrates that CD276 is broadly expressed on SCLC and significantly higher than of DLL3. This underscores the relevance of CD276 as a target antigen for immunotherapy in SCLC.

### T-cells express CD276 after stimulation, resulting in fratricide of CD276-directed CAR-T

**Fig. 2:**
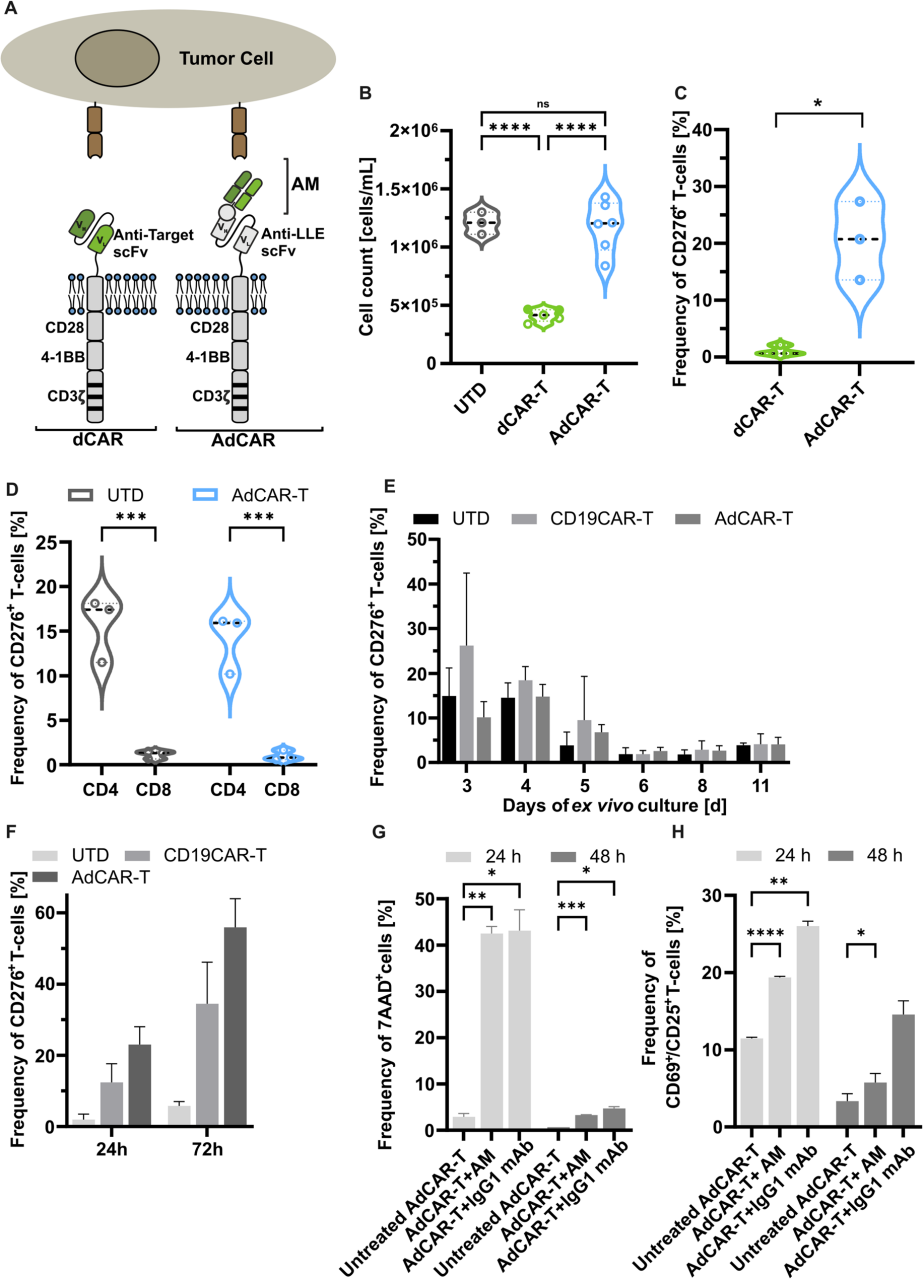
Upregulation of CD276 on activated CAR-T leads to decrease in cell number and fratricide in vitro. (**A**) Schematic overview of the AM and AdCAR system, as well as the d CAR. (**B**) dCAR-T culture at day six results in lower cell numbers compared to untransduced activated T-cells and AdCAR-T. n=6. ****p<0.0001 (One-way ANOVA). Error bars indicate SD between donors. (C) After six days of co-culture with SHP-77, dCAR-T show significantly less CD276-positive cells compared to AdCAR-T. n=3. *p<0.05 (paired t-test). Error bars denote SD between donors. (D) CD276 is mainly expressed on the activated CD4-positive fraction of AdCAR-T during in vitro culture and the fractions of CD4/CD8 show comparable CD276-expression to untransduced control T-cells. n=3. ***p<0.0005 (Two-way ANOVA). (E) During expansion of CD19-CAR-T and AdCAR-T ex vivo, the initial high expression of CD276 decreases over time. n=3. Error bars denote SD between donors. (F) CD276-expression on CD19CAR-T and AdCAR-T can be re-induced by stimulation with CD19-expressing target cells every 24 h. n=4. Error bars denote SD between donors. (G) In comparison to AdCAR-T only, AdCAR-T incubated with CD276-directed Fab or full mAb show and increased fraction of dead cells (7AAD+) as well as (H) a higher frequency of activated (CD69+/CD25+) cells. n=3. Error bars denote SD between donors. *p<0.05. **p<0.01. ***p<0.0005. ****p<0.0001 (Two-way ANOVA).

To target CD276 in SCLC, we designed a third generation CD276-directed CAR, incorporating CD28 and 4-1BB co-stimulatory and CD3-ζ signalling domains. In parallel, we evaluated our previously established third generation AdCAR platform (Fig. 2A) [20]. The AdCAR utilizes a specific scFv, which binds to biotin in the context of a specific linker, referred to as LLE. LLE-tags can be conjugated to mAbs or mAb fragments, generating AMs which mediate AdCAR-T to target antigen binding. We found impaired ex vivo expansion of dCAR-T in comparison to either un-transduced or AdCAR-transduced T-cells. On day six of CAR-T culture the absolute cell counts of dCAR-T were significantly lower compared to AdCAR-T or un-transduced T-cells, as demonstrated in six individual expansions from six individual donors (Fig. 2B). To study the underlying mechanisms, we determined the CD276-expression on dCAR-T and AdCAR-T by flow cytometry and saw a decreased CD276-expression on dCAR-T. In contrast, 20% of activated AdCAR-T and activated non-transduced T-cells, of those 15% CD4-positive T-cells, expressed CD276 (Fig. 2C-D). To understand the mechanisms leading to CD276-expression on T-cells, we stimulated freshly isolated human T-cells with either IL-7 and IL-15, OKT3, or TransAct (CD3 and CD28 agonistic signal). Consistently, CD276-expression was induced by the different T-cell activating stimuli (Additional file 2A), indicating a physiological response to universal T-cell activation. By concentrating on CD3/CD28 stimulation along with IL-7 and IL-15—the most commonly used method employed in CAR-T manufacturing—we observed a robust induction of CD276-expression in 10%-20% of T-cells across various conditions (CD19-CAR-T, AdCAR-T, and UTD) during the initial three days. This was followed by a steady decline in expression over the subsequent days of culture, a trend consistently observed in untransduced, AdCAR-transduced, and anti-CD19-CAR-Ts (Fig. 2E). Furthermore, CD276-expression on CAR-T can also be triggered by CAR-signalling alone, demonstrated for AdCAR-T directed against the antigen CD19 as well as antiCD19-CAR-T in repetitive co-culture with CD19-positive target cells (Fig. 2F). We hypothesise that impaired dCAR-T expansion results from either CD276-mediated tonic stimulation and subsequent exhaustion or fratricide. Comparing dCAR-T with AdCAR-T at day ten of ex vivo culture, we did not find significant differences in T-cell phenotype or expression of activation (CD69) and exhaustion markers (PD-1, Additional file 2B-C). CD276-negative dCAR-T can either result from fratricide of CD276-positive dCAR-T or blunting of the antigen by CAR-binding in cis. To address this question, activated AdCAR-T were treated with CD276-directed AMs. After 48 h of incubation, we found a significant increase in dead cells comparison to untreated AdCAR-T and significant increase in expression of activation markers on AdCAR-T in comparison to activated T-cells (Fig. 2G, H). No CD276-positive cells were detectable anymore (Additional file 2D). Together, the data strongly implicate that CD276-mediated fratricide in early dCAR-T culture results in impaired dCAR-T expansion and a suboptimal CAR-T product.

### Novel Fab-based AMs mediate specific lysis of CD276-positive SCLC tumor cells

While CD276-mediated fratricide occurs during ex vivo CAR-T manufacturing due to activation-induced expression, we hypothesized that it might also negatively impact CAR-T function during therapy, particularly under conditions of persistent CAR signalling. We therefore decided on developing Fab-based AMs to exploit the favourable pharmacokinetic properties of short serum half-life and, hence allowing resting periods of AdCAR-T [16]. We speculate that in the absence of AMs these resting periods not only limit CD276-mediated fratricide but also prevent AdCAR-T exhaustion.

**Fig. 3:**
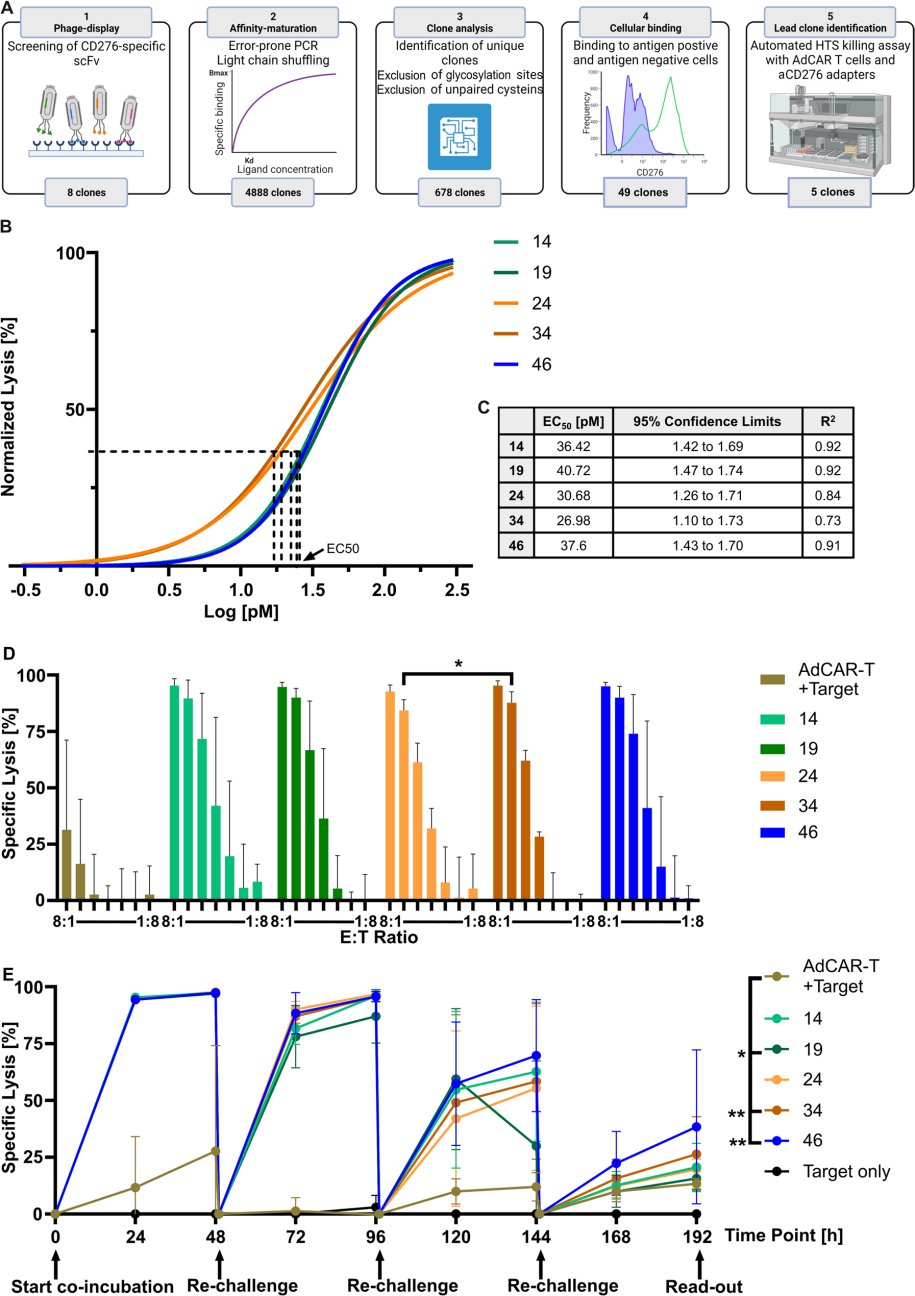
Novel biotinylated anti-CD276 AM mediates effective tumor cell elimination through AdCAR-T in vitro. (**A**) AM candidates were generated of parental clones through a naïve human phage display library screening. Through affinity maturation and exclusion of candidates with potential glycosylation sites and unpaired cysteines, unique clones were identified. Lead candidates were chosen based on manufacturability and specificity. (**B**) EC_50_ of the AM candidates in the AdCAR system calculated by linear regression and (**C**) the respective 95% confidence limits and R^2^. (**D**) Five novel AM candidates mediate specific cell lysis of the SCLC cell line SHP-77 by AdCAR-T at different E: T ratios after 48 h of co-incubation (5 nM AM). Negative lysis is a result of the calculation and equals target cell growth and is therefore excluded. *n* = 3. *p < 0.05 (Two-way ANOVA). Error bars indicate SD between donors. (**E**) Re-challenge cycles in which AdCAR-T were repeatedly exposed to SHP-77. AdCAR-T showed target cell lysis up to three re-challenge cycles and 192 h of co-culture (2 nM AM). Error bars denote SD between donors. *n* = 3. *p < 0.05. **p < 0.01 (Two-way ANOVA)

To generate novel CD276-directed AM candidates, a naïve human phage display library was screened for CD276-specific scFvs. Following an established workflow that included multiple rounds of antigen panning, scFv selection, affinity maturation, in silico prediction of manufacturability and analysis, transformation into Fab format, and functional testing for manufacturability and specificity, five lead candidates were identified. These candidates were also evaluated for their capability to mediate AdCAR-T activation (Fig. 3A and Additional file 3A-E). The five generated anti-CD276 AMs mediate highly specific target cell lysis by AdCAR-T at picomolar concentrations, with an EC_50_ ranging from 27 to 41 pM and even at low E: T ratios (Fig. 3B and C). When cultivated with SCLC tumor cells at different E: T ratios, the AM46 specifically mediated target cell lysis (Fig. 3D). AdCAR-T were able to repeatedly lyse SHP-77, in up to three re-challenge cycles. After the third cycle and 192 h of co-incubation, AM46 was found to still mediate lysis of over 25% SHP-77 cells, significantly outperforming the other tested AMs (Fig. 3E). The performance of the AMs was tested in additional CD276-positive cell lines from various cancer entities, confirming the high efficacy of AM46 (Additional file 4 A-C). Additionally, cytokine levels (GM-CSF, IFN-γ and TNF-α) during re-challenge with SHP-77 were analysed and no significant differences in cytokine production were found across the tested AMs (Additional file 4 D-F). In summary, functional in vitro testing of all five developed AMs demonstrated specific activation of AdCAR-T and highly efficient lysis of CD276-positive target cells. Due to the favourable manufacturing properties (e.g. production yield) and high functional performance, AM46 was selected for downstream in vivo evaluation.

### AdCAR-T induce remission and control SCLC progression in vivo

**Fig. 4:**
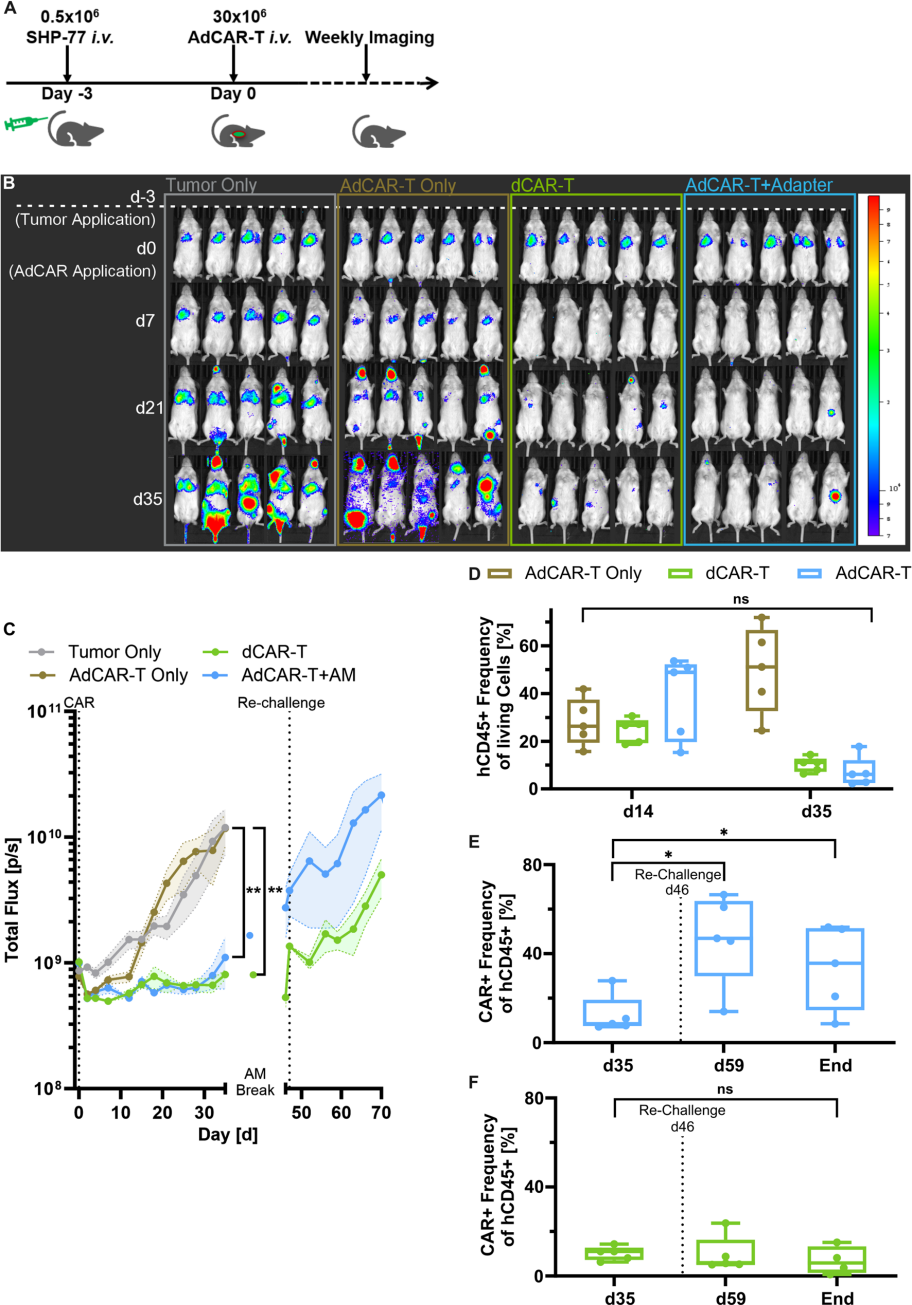
AdCAR-T can actively control disease progression of SCLC in vivo. (A) Schema of in vivo experiment using 0.5*10^6 SHP-77 tumor cells, engrafted into the lungs, and challenged by 30*10^6 AdCAR-T in combination with the AM46 (100 µg). (B) Representative BLI images of the SHP-77 mouse model on day -3, 0, 7, 21, and 35. Color scale represents radiance from 7x103 to 9x104 photons/sec. Mice were sacrificed when reaching termination criteria approx. at a total flux [p/s] of 1010. (C) Representative BLI kinetics of tumor signal of the mice receiving the different treatment options (tumor only, AdCAR-T only, dCAR-T and AdCAR-T+AM) of the respective imaging days. **p<0.01 (Mixed-effects Model with Geisser-Greenhous correction). (D) Whole mouse blood of AdCAR-T only, dCAR-T and AdCAR-T without AM46, analysed on day 14 and 35 for the frequency of human CD45-positive cells. Dead and murine cells were excluded prior. n=5. SD between mice is indicated by error bars (paired t-test). CAR-T frequency of mice treated with (E) AdCAR-T+AM46 or (F) dCAR-T in whole mouse blood on day 35, on day 59 and at the End. Re-challenge was done on day 46 with 0.5x106 SHP-77. n=5. SD between mice is indicated by error bars. *p<0.05 (Wilcoxon test).

To test in vivo activity of CD276-directed AdCAR-T against SCLC, a disseminated xenograft model of SCLC was established. eFFly-expressing SHP-77 cells were injected intravenously into mice and orthotopic engraftment to the lungs was verified by BLI. Mice were randomized and treated with either dCAR-T, AdCAR-T only without AM46, or AdCAR-T plus AM46. AM46 was administered intraperitoneally once per day (Fig. 4A). In contrast to untreated AdCAR-T and AdCAR-T without AM46 group, both dCAR-T and AdCAR-T plus AM46 achieved initial remission and prolonged disease control (Fig. 4B and C). In is noticeable that the fraction of CD4-positive cells is in dCAR-T treatment group significantly lower compared to the AdCAR-T group (Additional file 5 C). Monitoring of CAR-T in peripheral blood revealed an early expansion, followed by a reduction of dCAR-T and AdCAR-T in the AdCAR-T plus AM46 group. In contrast, AdCAR-T only without AM46, while not mediating specific tumor lysis, further expanded over time (Fig. 4D). These results underscore that targeting CD276 interferes with CAR-T expansion and persistence. After day 35 post CAR-T application, AM46 dosing was paused, resulting in disease progression in the AdCAR-T plus AM46 group (Fig. 4E). These findings suggest that disease is actively controlled until day 35 by AdCAR-T and requires the presence of the AM46 for disease control. To test functional persistence of CAR-T, mice were re-challenged with SHP-77 cells, administered intravenously on day 46. Even though tumor load in the AdCAR-T plus AM46 group at this timepoint was significantly higher in comparison to the dCAR-T group, complete and partial responses were achieved after re-challenging and re-initiation of AM46 application, clearly demonstrating functional persistence of AdCAR-T (Additional file 5 A). Moreover, AdCAR-T supported robust expansion after re-challenging and “reactivation” in comparison to dCAR-T (Fig. 4E-F). Together, we clearly demonstrate in vivo activity in both CD276-targeted AdCAR-T. Importantly, AdCAR-T retain fitness and capability to expand after AM46 pausing, while dCAR-T showed inferior performance.

### Optimized dosing of AMs improves AdCAR-T expansion and functional persistence

**Fig. 5:**
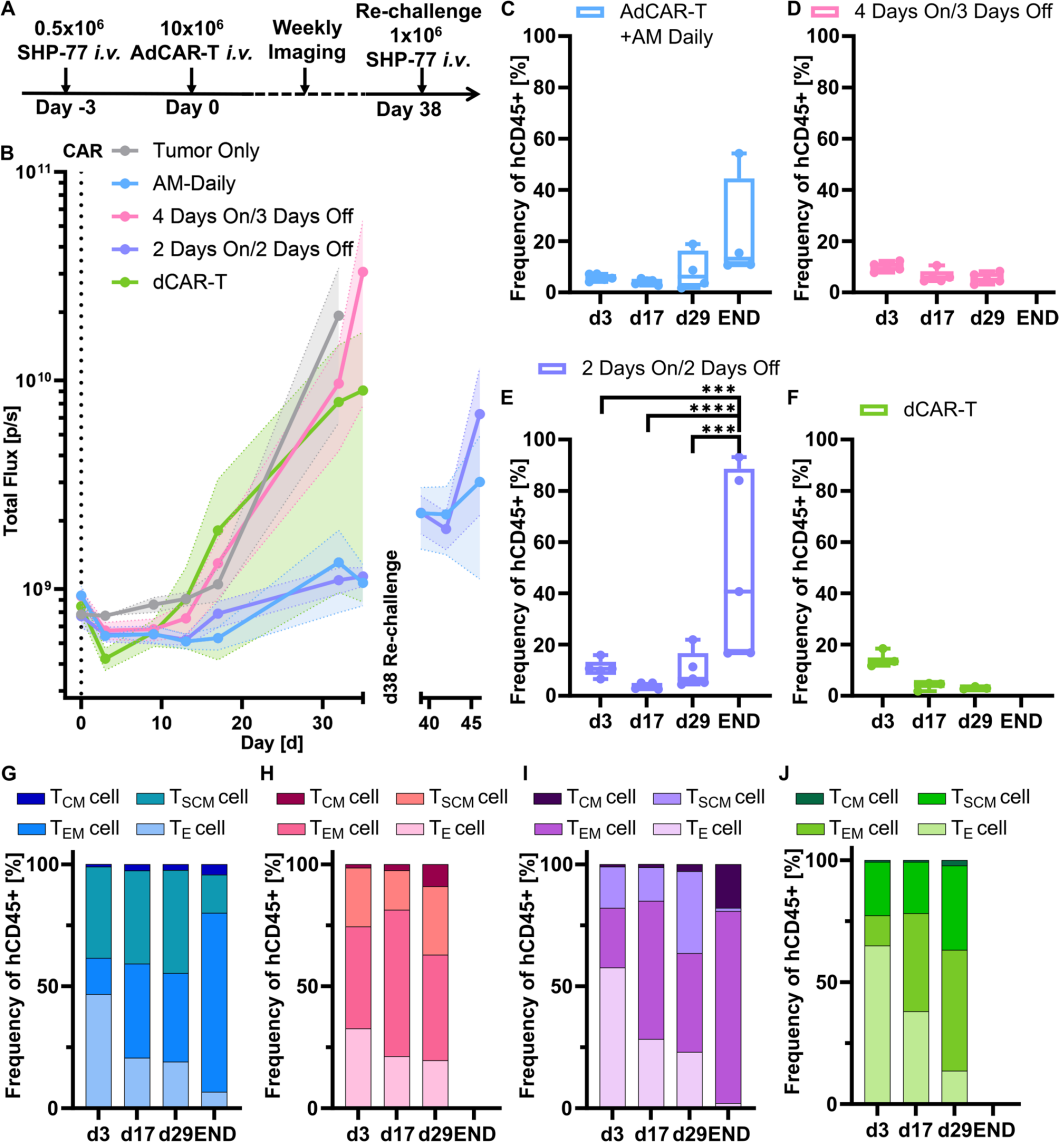
Dosing scheme enhances T-cell expansion after re-challenge, but daily dosing shows efficient anti-tumor effect. (**A**) Mice were ingrafted with SHP-77 cells and three days later infused with AdCAR-T. Re-challenge with 1 × 10^6^ SHP-77 at day 38. AM46 dosing either daily (Cond I), four days on / three days off (Cond II), or Two days on, two days off (Cond III). (**B**) BLI kinetics of the mouse groups receiving AdCAR-T treatment plus AM46 in different dosing schemes, and a re-challenge with SHP-77 on day 38. Whole mouse blood was analysed for the frequency of human CD45-positive cells on day 3, 17, 29, and at the End of the treatment groups: (**C**) dosing regimen I: AdCAR-T with AM46 daily administration; (**D**) dosing regimen II: 4 days AM46 administration, followed by 3 day break; (**E**) dosing regimen III: 2 day AM46 administration, followed by 2 day break and (**F**) condition IV: dCAR-T. *n* = 5. ***p < 0.0005. ****p < 0.0001 (Mixed-effects model). T-cell phenotypes of differentiation subsets in (**G**) dosing regimen I, (**H**) dosing regimen II, (**I**) dosing regimen III or (**J**) condition IV: dCAR-T were analysed. *n* = 5. **p < 0.01 (Two-way ANOVA)

Having demonstrating improved expansion after AdCAR-T resting periods (Fig. 4), we aimed to optimize AM46 dosing schedules. To detect subtle differences in functional performance, the in vivo model was adjusted to a “stress test” design (Fig. 5A). For this, the amount of administered CAR-T was reduced to half, but doubled the tumor burden was administered during the re-challenge (Fig. 5A). Three different AM46 dosing regimens were compared: Dosing regimen I: daily administration, dosing regimen II: two days with AM (´on´) / two days without AM (´off´) and dosing regimen III: four days on/three days off. In the adjusted “stress test” setting, dCAR-T, serving as a control, initially induced remission, however the disease rapidly progressed thereafter (Fig. 5B). Consistently, no circulating dCAR-T were detectable in the blood on day 29 (Fig. 5C). Dosing regimen III failed to induce lasting disease control, further supporting the hypothesis that continuous AdCAR-T stimulation and target-directed activity is required. In contrast, both dosing regimens I and II resulted in extended disease control and transient re-induction of T-cell expansion after re-challenge with SHP-77 cells (Fig. 5C-F). Notably, AdCAR-T frequency in peripheral blood after re-challenging were higher in dosing regimen II, suggesting improved T-cell fitness. T-cell phenotyping revealed a larger proportion of central memory T-cells (T_CM_) in dosing regimen II in contrast to dosing regimen I, indicating direct effects of daily AdCAR-T stimulation on T-cell differentiation states (Fig. 5G-J). Against the hypothesis that extended “resting” periods improve CAR-T function, daily AM46 dosing was shown to outperform other evaluated dosing schemes in terms of disease control. This may be due to the very aggressive tumor model, which requires continuous therapeutic pressure. In summary, disease control was best with dosing regimen I. However, dosing regimen II resulted in beneficial expansion potential after re-challenging and shifting T-cell differentiation states.

### Activation-induced IL-18 secretion further enhances AdCAR-T function

**Fig. 6:**
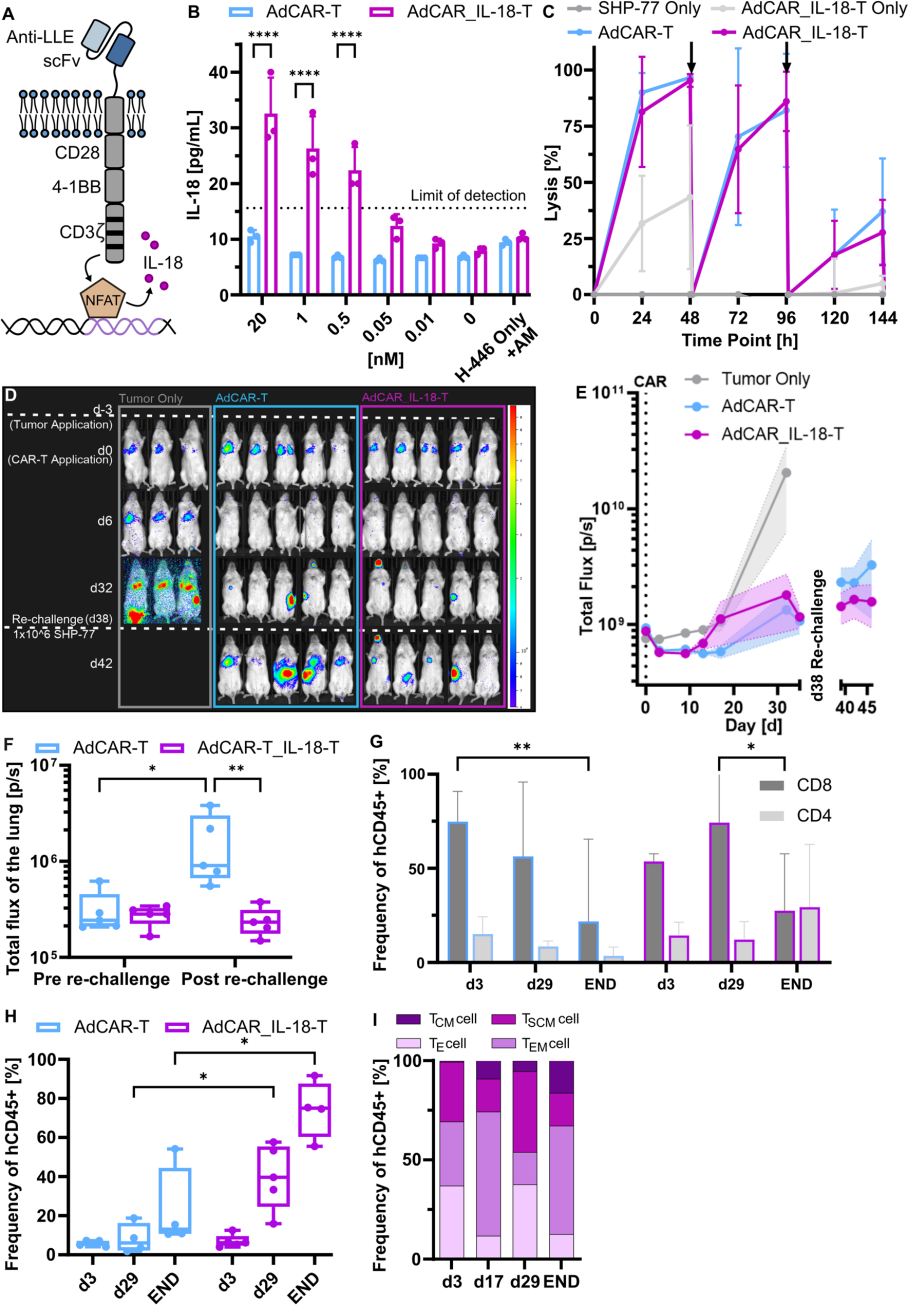
AdCAR-T loaded with IL-18 enhance T_CM_ differentiation, cell expansion, and anti-tumor effect. (A) Schematic overview of the TRUCK AdCAR-T construct. Binding of the AdCAR-T to its target mediated by AM46 leads to activation of NFAT. NFAT induces the secretion of IL-18 by the cell. (B) IL-18 secretion of AdCAR_IL-18-T upon co-incubation with H-446. n=3. SD is indicated by Error bars. ****p<0.0001 (Two-way ANOVA). (C) Specific lysis of AdCAR_IL-18-T co-incubated with SHP-77 in vitro and re-challenged every 48 h, as indicated by arrows. n=3. SD is denoted by error bars (Two-way ANOVA). (D) Representative BLI mouse images of the groups receiving no CAR-T, AdCAR-T or AdCAR_IL-18-T plus daily AM46. Images taken on day 0, 6, 32, and on day 42 after re-challenge with 0.5x106 SHP-77. (E) BLI kinetics of mice treated with either AdCAR-T or AdCAR_IL-18-T with daily AM46. Re-challenge was performed at day 38. (F) BLI analysis of the lungs of mice receiving either AdCAR-T or AdCAR_IL-18-T with daily dosing of AM46 before and after re-challenge with SHP-77. n=5. *p<0.05. **p<0.01 (Two-way ANOVA). (G) Proportion of CD4-positive T-cells and CD8-positive T-cells of all CD45+ T-cells of the mice treated with AdCAR-T and AdCAR_IL-18-T. Depicted on day 3, day 29 and the End. n=5. SD between mice is indicated by error bars. *p<0.05 (Two-way ANOVA). Error bars indicate SD between mice. (H) Whole mouse blood of the AdCAR-T and AdCAR_IL-18-T groups were analysed for the frequency of human CD45-positive cells on day 3, 29, and at the End, showing higher frequencies for AdCAR_IL-18-t at d29 and at the End. n=5. **p<0.01 (Two-way ANOVA). (I) Flow cytometry analysis of T-cell subsets of the AdCAR_IL-18-T treatment group and frequency of T-cells on day 3, 17, 29, and at the End. n=5. *p<0.05 (Two-way-ANOVA).

Induced IL-18 secretion has been demonstrated to improve effecter function and anti-tumor activity of CAR-T against solid cancers [32, 33]. To evaluate whether IL-18 secretion can further enhance AdCAR-T function in combination with our novel CD276-targeting AM46, a vector co-expressing IL-18 was designed (Fig. 6A) [34]. Therefore, human IL-18 was placed after an 3´ NFAT-inducible NFATsyn promotor containing six consensus NFAT enhancer repeats and a synthetic TATA box, followed by an PGK promotor driving constitutive AdCAR expression. To demonstrate IL-18 secretion induced by target specificity and AdCAR-activity, AdCAR_IL-18 was expressed in human T-cells. AdCAR_IL-18-T and AdCAR-T were co-cultured with CD276-positive target cells, testing increasing doses of AM46. IL-18 secretion above baseline was only detected in the supernatant of AdCAR_IL-18-T in an AM-dose dependant manner (Fig. 6B). In vitro, killing capacity after repetitive stimulation with tumor cell lines was comparable between AdCAR_IL-18-T and AdCAR-T (Fig. 6C). For the in vivo analysis, mice were engrafted with SHP-77 cells and treated with AdCAR-T or AdCAR_IL-18-T together with daily AM46 administration (dosing regimen I in Fig. 5). We did not see significant differences between AdCAR_IL-18-T and AdCAR-T in tumor control over the first 32 days. However, AdCAR_IL-18-T demonstrated improved anti-tumor activity after re-challenge (Fig. 6D and E). Analysis of BLI signals revealed a significant reduction in the lung region after re-infusion of SCLC cells, indicating targeted disease clearance (Fig. 6F). Whole blood analysis showed a significant increase in T-cell counts in AdCAR_IL-18-T compared to AdCAR-T at day 29 and after the re-challenge, indicting a positive effect of IL-18 on proliferation and functional persistence. Particularly, the persistence of CD4-positive T-cells was promoted in the AdCAR_IL-18-T group, resulting in an equal CD4:CD8 ratio after re-challenge. In the AdCAR-T group, the number of CD8-positive cells was seven times higher than the frequency of CD4-positive cells (Fig. 6G-H). Furthermore, AdCAR_IL-18-T supports differentiation into T_CM_ and T_EM_ (Figs. 5G and 6I). Together, AdCAR_IL-18-T are able to impede SCLC growth and prevent the engraftment of the SHP-77 cells in the lungs after re-challenge. These results suggest AdCAR_IL-18-T plus AM46 (daily or two days/on two days off) as the lead combination for further clinical development.

## Discussion

CD276 is an emerging target antigen, expressed on various solid tumor entities. Due to its expression on cancer-initiating cells and tumor vasculature, it could become an effective therapeutical target to disrupt TME-barriers, alongside targeting the tumor cells directly [23, 35]. In the present study, we explored CD276-expression on SCLC and its potential usage for immunotherapeutic intervention. We found homogeneous expression across the SCLC subtypes, both at RNA and protein level, as it is described in the literature [36]. CD276 showed a strong membrane-associated expression suitable for mAb or CAR-T targeting. Its expression was found to be significantly higher compared to DLL-3, currently the first-in-class antigen target in SCLC [37], making CD276 a compelling alternative.

To target CD276 with immunotherapy in SCLC, we evaluated two different CAR-constructs, which differ in their mechanism of tumor targeting: dCAR-T, which directly target CD276; and AdCAR-T, which indirectly target CD276 via an AM. Multiple studies have described the development and preclinical evaluation of CD276-targeting CAR-T and recently the first clinical data for aCD276-CAR-T in solid tumors and CNS malignancies have been reported [24, 25, 38–40]. While demonstrating safety of both intravenous and intrathecal administration, the clinical efficacy was limited, partially due to poor CAR-T expansion and persistence within the patients. Consistently, we found rapid decline of directly targeting CAR-T frequencies in SCLC in vivo models. Moreover, the dCAR-T showed impaired ex vivo expansion in comparison to non-transduced T-cells or AdCAR-transduced and activated T-cells. We show that CD276 is expressed on activated CAR-T, with a notably high expression observed in the activated CD4-positive cell subset. Our findings demonstrate that this CD276-expression on T-cells is induced by various activating stimuli, including CD3 and/or CD28 stimulation, CAR signalling, and by activating cytokines such as IL-7 and IL-15. This aligns with previous research identifying CD276 as a marker on activated or alloreactive T-cells, particularly with elevated expression in activated CD4-positive subsets [41]. Consequently, the pronounced CD27-expression on CAR-T may heighten the risk of graft-versus-host disease (GVHD). Furthermore, in vitro expression of CD276 on CAR-T can lead to self-targeting, particularly if the CAR-T construct is designed to target CD276 (as with dCAR-T). This phenomenon, known as fratricide, can result in restricted CAR-T expansion and diminished activity.

In order to address this limitation, we utilized our AdCAR-T platform, in which the tumor targeting is mediated by an interposed AM. To develop an optimized AM, we first performed a comprehensive phage library screen to identify novel CD276 binder with ideal binding properties. The resulting lead candidates went through affinity maturation and extensive functional in vitro testing. This resulted in an AM with high *in* vitro functionality and good properties for manufacturing. The format of the AM is Fab-based, which allows to mitigate fratricide and prevent overstimulation due to its short serum half-life, resulting in rapid AM clearance and temporally limited AdCAR-T activity. During the ex vivo cell expansion, the AdCAR-T benefited from the lack of CD276 targeting. This supported robust cell expansion and preserved a central memory phenotype of the AdCAR-T, which is associated with high antitumoral efficacy and long-time survival [42, 43]. Back-to-back testing of the optimized aCD276-AMs with AdCAR-T in a disseminated stress-test in vivo model of SCLC demonstrated superior anti-tumor activity and functional persistence of AdCAR-T after tumor re-challenging, compared to dCAR-T. We have previously demonstrated that intermittent or “paused” AM dosing can prevent AdCAR-T exhaustion caused by continuous stimulation and that fluctuating AM serum levels resulting from daily dosing might moderate exhaustion [35]. In contrast to our studies in AML, the SCLC model did not allow prolonged intervals between AM administration due to rapidly progressing disease, highlighting the need for an active and continued control of malignant cells by AdCAR-T. These findings support the rationale for applying this approach in settings of low disease burden, such as MRD, to prevent relapse. This is in line with potential use in the adjuvant setting, where effective post-remission therapies are urgently needed to improve overall survival in SCLC.

IL-18 is a cytokine which plays a major role in IFN-γ secretion and stimulation of the innate immune system [44–46]. Integrating IL-18 into CAR-T resulted in increased anti-tumor function, cytokine secretion, survival and T-cell persistence in several studies [45, 47–49]. To transfer this amplification of desired properties to our system, AdCAR-T of this study was enriched with a NFAT-inducible IL-18 cassette to generate a 4th generation CAR-T. In vitro and in vivo analysis showed significantly improved cell expansion and the differentiation to a favourable T-cell phenotype such as T_SCM_. Additionally, the secretion of IL-18 of the AdCAR-T resulted in a high frequency of CD4-positive CAR-T, which is typically associated with long-time persistence of CAR-T and high remission rates in patients [50, 51]. This was also confirmed by the prolonged persistence of IL-18 armoured AdCAR-T in mice blood. While the fundamental concept of TRUCK-CAR-Ts, particularly IL-18 secreting CAR-T targeting SCLC, is not novel, this represents the first demonstration of AM-dependent remote-controlled IL-18 secretion by CAR-T.

Currently, upon newly diagnosed SCLC and relapsed SCLC, the standard clinical practice of SCLC is the administration of various checkpoint inhibitors such as Atezolizumab (PD-L1) or Ipilimumab (CTLA-4), together with radiotherapy and platinum-based chemotherapy [2]. Even though this has proven to prolong the overall survival of the patients, most suffer from severe side effect and suffer from immunotoxicities. In contrast, our AdCAR-T offers a controlled and targeted approach that specifically attacks cancer cells by recognizing unique antigens on their surface, potentially sparing healthy cells and reducing the associated side effects. Additionally, our system showed a durable response with elevated levels of CD4-positive T-cells and the differentiation into durable T_SCM_/ T_CM_, opening up the possibility of persistent tumor control and sustained remission in the patients. As a next step and to address tumor heterogeneity combinatorial antigen targeting in a PDX model could be further explored.

## Conclusion

In conclusion, we have demonstrated a high prevalence of CD276 expression on primary human SCLC specimens. We describe the development of a novel, optimised Fab-based AM that targets CD276 and attenuates CD276-dependent fratricide. In an in vivo model of disseminated SCLC, tailored AM dosing and cytokine support through an activation-induced IL-18 armour approach resulted in improved functional persistence and anti-tumour activity of AdCAR-T. Taken together, our results suggest that AdCAR-T is ideally suited to target CD276 in SCLC. Despite its expression on activated T-cells, CD276 remains a highly promising therapeutic target due to its broad and uniform expression across multiple malignancies. Innovative strategies such as controlled adapter dosing, as demonstrated in this study, or gene editing approaches may provide effective means to overcome CD276-dependent fratricide and maximize therapeutic efficacy. We plan to test our system in a Phase I/II clinical trial evaluating CD276-targeting AdCAR-T in SCLC in patients with complete (CR1) or partial remission (PR1).

## Supplementary Information

Below is the link to the electronic supplementary material.

Additional file 1: Exemplary gating strategy for AdCAR-T (**A**) first cell population gated. Then dubplates were excluded based on SSC and FSC parameters. Next, dead cell were excluded. At last CAR-T population was identified

Additional file 2: Analysis of characteristics of ex vivo CAR-T (**A**) CD276-expression is upregulated upon stimulation of T-cells with either, IL-7/IL-15, OKT3, or TransAct. (**B**) Frequency of the T_CM_cell, T_SCM_cell, T_EM_cell and T_E_cell subtype after 10 days of *in vitro* cell culture. (**C**) Frequency of CD69-positive and PD-1-positive CD19-CAR-T and AdCAR-T is comparable to untransduced T-cells after 10 days of *in vitro* cell culture. (**D**) CD276-expression decreases on AdCAR-T co-cultured with CD276-directed Fab or full mAb after 2 h of culture. n = 3. Error bars depict SD between replicates. *p < 0.05. **p < 0.01. ****p < 0.0001 (paired t-test)

Additional file 3: Development of novel AMs From the phage library screen, the Fab-based AM candidates were identified. To test them, (**A.1**) a high throughput screening was conducted, where AdCAR-T, directed against a tag on the newly generated binders, were repeatedly exposed to OCI-AML-2 to test the tumor cell lysis. The Fab based AM lead-candidates, indicated with a red arrow, mediate the highest tumor cell lysis by AdCAR-T. In contrast, the control condition (**A.2**) without AdCAR-T show no unspecific tumor cell lysis. AM candidates are depicted on the x-axis. n = 3. SD between technical replicates indicated by error bars. (**B**) After affinity maturation, 41 Fabs with high binding specificity to CD276-expressing cells (y-axis) and no unspecific binding (x-axis) were chosen for further functional testing with AdCAR-T. (**C**) The five lead candidates from HTS were analysed for their binding specificity on OCI-AML-2 cells via flow cytometry and (**D**) for their manufacturability (protein yield and thermostability)

Additional file 4: Analysis of the specific cell lysis of AdCAR-T with the novel AMs Specific lysis of (**A**) RH30, (**B**) IMR32, and (**C**) DMS114 as target cells with AdCAR-T plus the five AM candidates. Re-challenge was performed every 48 h with the initial tumor cell count. Significance is depicted from end time point (192h). Error bars denote SD between donors. n = 3. *p < 0.05. ****p < 0.0001 (Mixed-Effect Model). (**D-F**) Cytokines (IFN-γ, GM-CSF and TNF-α) secreted by the AdCAR-T at 24 h, 48 h, and 96 h of co-culture with antigen expressing SHP-77 cells and AM46. Error bars denote SD between donors. n = 3. *p < 0.05. **p < 0.01. ****p < 0.0001 (Mixed-Effect Model). (**G**) Frequency of LAG3 and PD-1 double positive AdCAR-Ts after 192h of co-culture with the AM candicatesand four re-challenge cycles with SHP-77. Error bars denote SD between donors. n = 3 (Two-way ANOVA)

Additional file 5: In vivo analysis of AcCAR-T + AM46 (**A**) Representative mouse images of the groups receiving no CAR-T, AdCAR-T without AM, dCAR-T or AdCAR-T with AM46. Images are on day 35 and 46 before re-challenge with 0.5x10^6^ SHP-77. Continuing with images from after re-challenge on day 47, 52, 59 and 70. n = 5. (**B**) Representative BLI kinetics of tumor signal of the mice with the different treatment groups on the respective imaging days. AM dosing breaks occurred between day 35 and 46. Re-challenge of the mice with 0.5x10^6^ SHP-77 occurred on day 46. Color scale represents radiance from 3x10^4^ to 1x10^6^ photons/sec. SD between mice is indicated by error bars. n = 5. **p < 0.01 (Mixed-Effect Model). (**C**) Analysis of the fraction of hCD45+/CD4+ cells of the treatment groups dCAR-T and AdCAR-T + AM daily on day 3 after *in vivo* injection into the mouse model. n = 3 for dCAR-T. n = 5 for AdCAR-T + AM Daily. *p < 0.05 (Unpaired t-test)

Additional Table 1: Comprehensive list of the used antibodies with their respective fluorochrome, manufacturer, clone, dilution and identifier

Additional Table 2: Used reagents with their respective manufacturer, dilution and identifier

## Data Availability

Datasets generated and/or analysed during the current study are available from the corresponding author on reasonable request. For original data, please contact christian.seitz@med.uni-heidelberg.de.

## Abbreviations

aCD276: Against CD276
aCD276-CAR-T: Against CD276 targeting CAR-T cells
AdCAR-T: Adapter-CAR T-cells
AdCAR-T_IL-18: AdCAR-T-cells encoding IL-18
AM: Adapter molecule
B-ALL: B-cell Acute lymphocytic leukaemia
CAR: Chimeric antigen receptor
CAR-T: Chimeric antigen receptor T-cells
CD19-CAR-T: Directly CD19-targeting CAR-T
CR1: Complete remission
dCAR-T: Directly CD276-targeting CAR-T
E:T: Effector to target ratio
eFFly: Firefly luciferase
Fab: Fragment antigen binding
FFPE: Formalin-fixed and paraffin-embedded
GVHD: Graft-versus-host disease
H: Hours
ICB: Immune checkpoint blockade
LLE: Linker-label epitope
LV: Lentiviral vector
mAb: Monoclonal antibody
min: Minutes
MM: Multiple myeloma
NHL: Non-Hodgkin lymphoma
NSCLC: Non-small cell lung cancer
PR1: Partial remission
sAdCAR-T: Tag directed AdCAR-T
scFv: Single chain variable fragment
SCLC: Small cell lung cancer
T_CM_: Central memory T-cell
T_E_: Effector T-cell
T_EM_: Effector memory T-cell
TMA: Tissue microarray
TME: Tumor micro environment
T_SCM_: Stem cell-like memory T-cell
