# Supplementary figures and images for: Targeting CD276 with Adapter-CAR T-cells provides a novel therapeutic strategy in small cell lung cancer and prevents CD276-dependent fratricide
Source: J Hematol Oncol. 2025 Jul 28;18:76. doi: 10.1186/s13045-025-01729-8 (PMC12305915; doi:10.1186/s13045-025-01729-8)

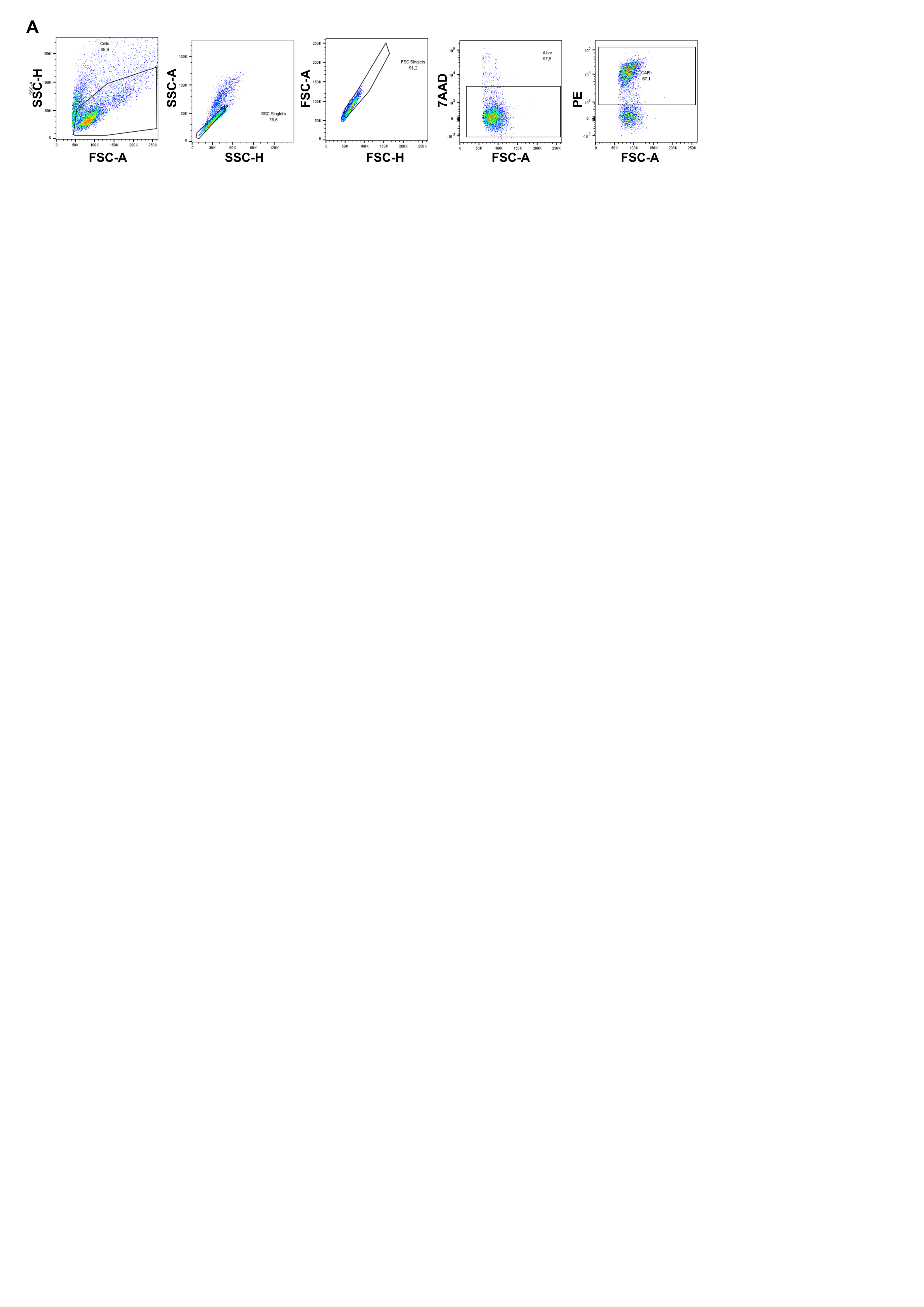

Supplement: Supplementary file 1 — Additional file 1: Exemplary gating strategy for AdCAR-T (A) first cell population gated. Then dubplates were excluded based on SSC and FSC parameters. Next, dead cell were excluded. At last CAR-T population was identified [file 13045_2025_1729_MOESM1_ESM.tiff]

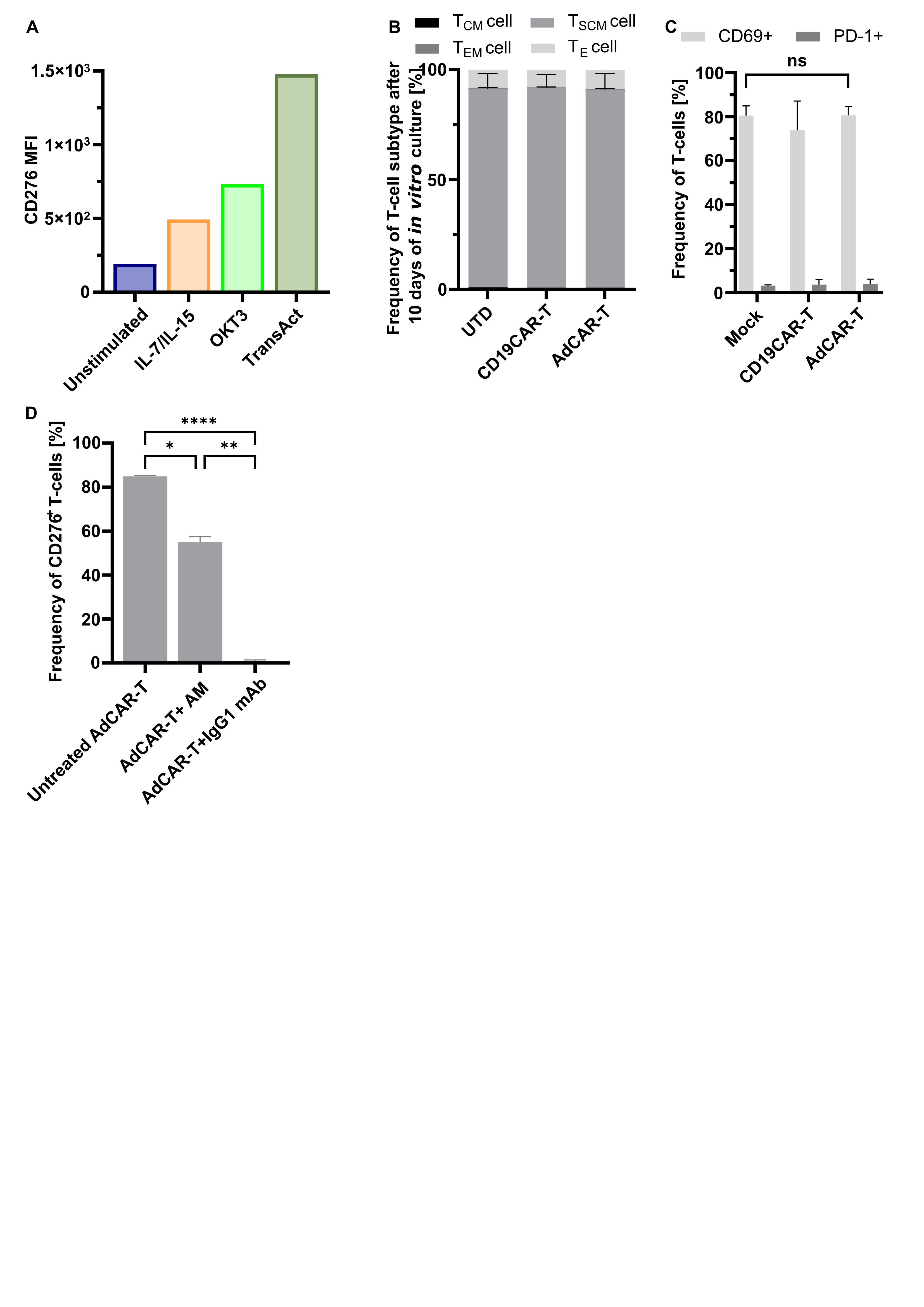

Supplement: Supplementary file 2 — Additional file 2: Analysis of characteristics of ex vivo CAR-T (A) CD276-expression is upregulated upon stimulation of T-cells with either, IL-7/IL-15, OKT3, or TransAct. (B) Frequency of the TCMcell, TSCMcell, TEMcell and TEcell subtype after 10 days of in vitro cell culture. (C) Frequency of CD69-positive and PD-1-positive CD19-CAR-T and AdCAR-T is comparable to untransduced T-cells after 10 days of in vitro cell culture. (D) CD276-expression decreases on AdCAR-T co-cultured with CD276-directed Fab or full mAb after 2 h of culture. n = 3. Error bars depict SD between replicates. *p < 0.05. **p < 0.01. ****p < 0.0001 (paired t-test) [file 13045_2025_1729_MOESM2_ESM.tiff]

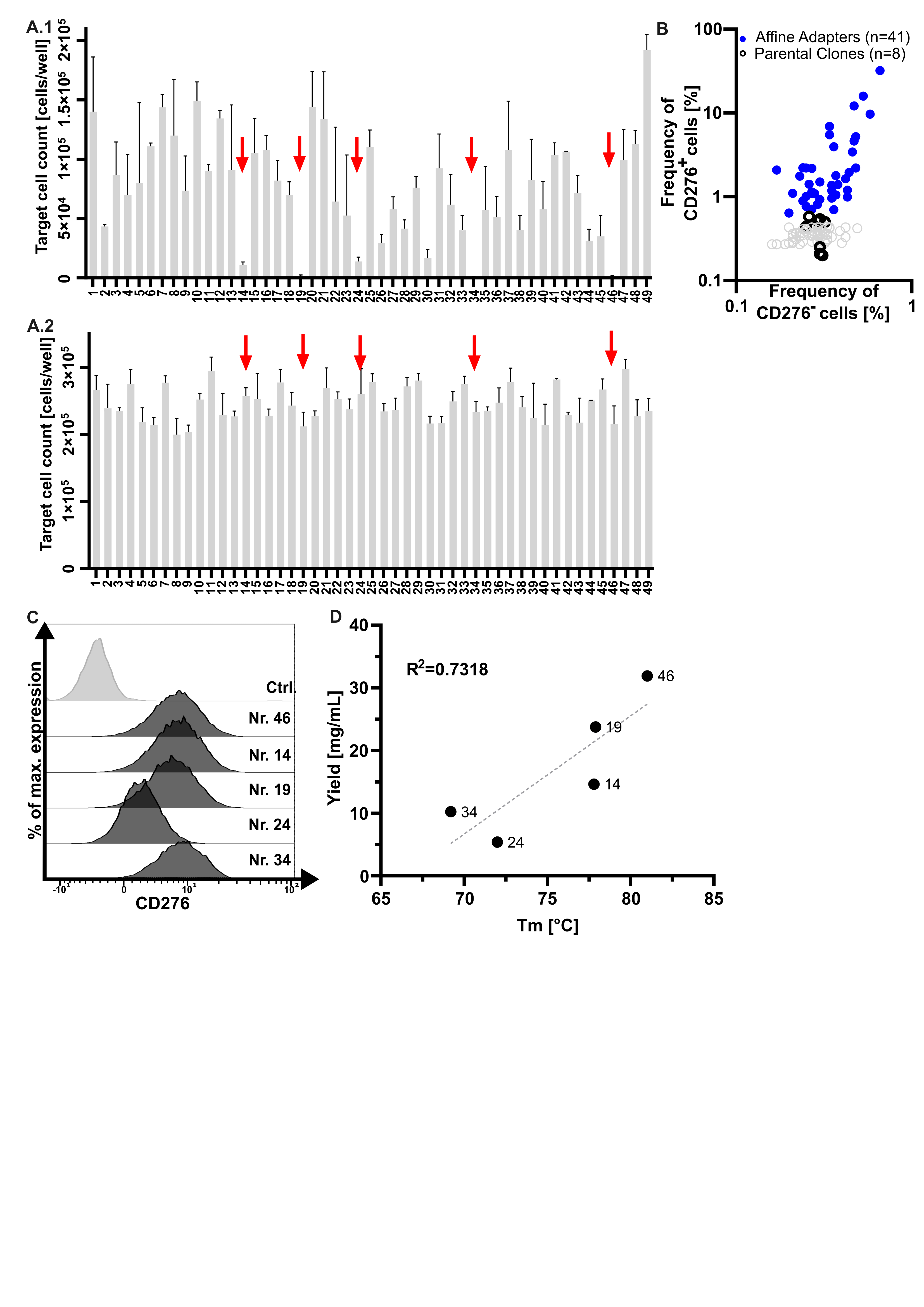

Supplement: Supplementary file 3 — Additional file 3: Development of novel AMs From the phage library screen, the Fab-based AM candidates were identified. To test them, (A.1) a high throughput screening was conducted, where AdCAR-T, directed against a tag on the newly generated binders, were repeatedly exposed to OCI-AML-2 to test the tumor cell lysis. The Fab based AM lead-candidates, indicated with a red arrow, mediate the highest tumor cell lysis by AdCAR-T. In contrast, the control condition (A.2) without AdCAR-T show no unspecific tumor cell lysis. AM candidates are depicted on the x-axis. n = 3. SD between technical replicates indicated by error bars. (B) After affinity maturation, 41 Fabs with high binding specificity to CD276-expressing cells (y-axis) and no unspecific binding (x-axis) were chosen for further functional testing with AdCAR-T. (C) The five lead candidates from HTS were analysed for their binding specificity on OCI-AML-2 cells via flow cytometry and (D) for their manufacturability (protein yield and thermostability) [file 13045_2025_1729_MOESM3_ESM.tiff]

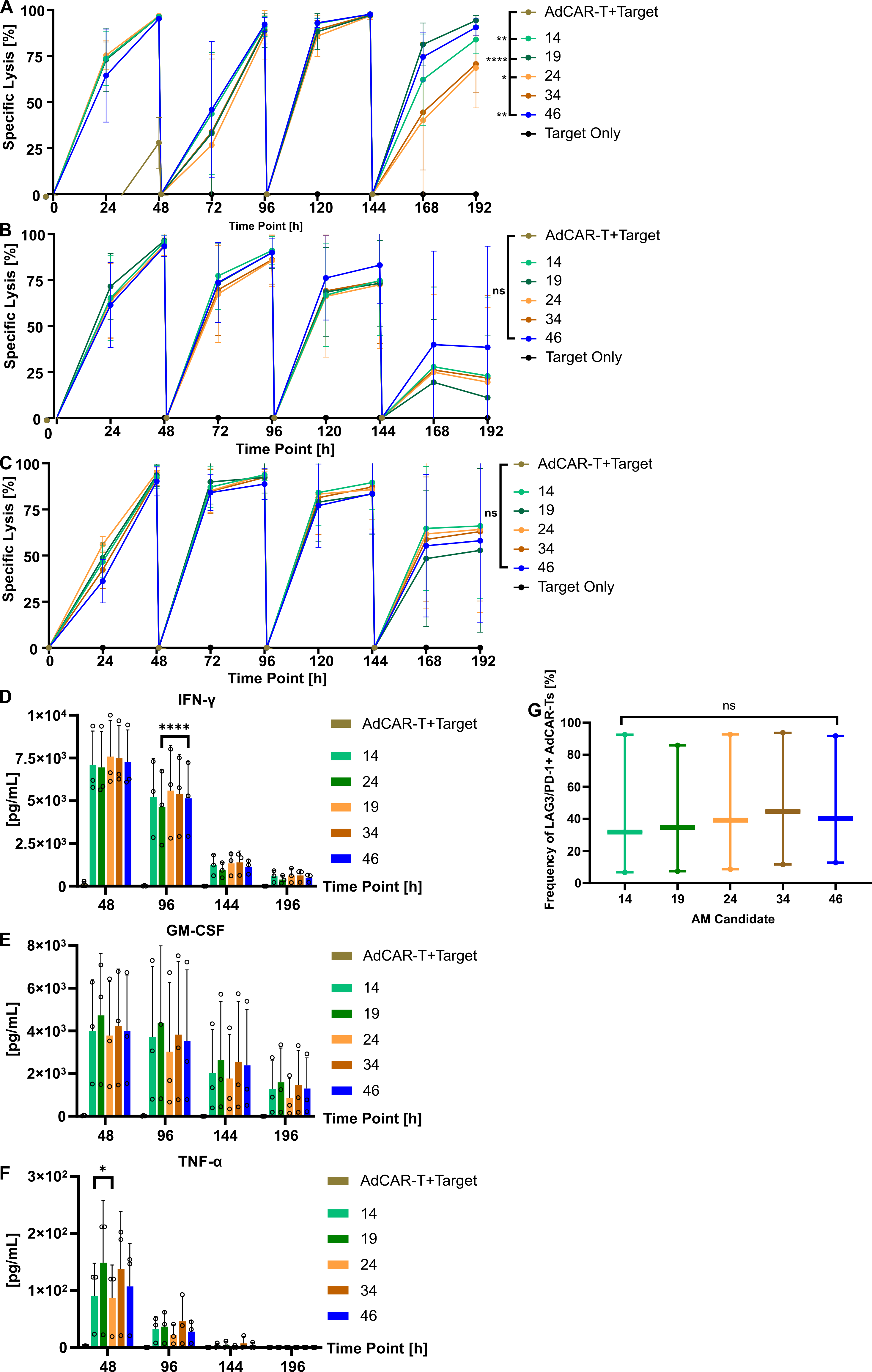

Supplement: Supplementary file 4 — Additional file 4: Analysis of the specific cell lysis of AdCAR-T with the novel AMs Specific lysis of (A) RH30, (B) IMR32, and (C) DMS114 as target cells with AdCAR-T plus the five AM candidates. Re-challenge was performed every 48 h with the initial tumor cell count. Significance is depicted from end time point (192h). Error bars denote SD between donors. n = 3. *p < 0.05. ****p < 0.0001 (Mixed-Effect Model). (D-F) Cytokines (IFN-γ, GM-CSF and TNF-α) secreted by the AdCAR-T at 24 h, 48 h, and 96 h of co-culture with antigen expressing SHP-77 cells and AM46. Error bars denote SD between donors. n = 3. *p < 0.05. **p < 0.01. ****p < 0.0001 (Mixed-Effect Model). (G) Frequency of LAG3 and PD-1 double positive AdCAR-Ts after 192h of co-culture with the AM candicatesand four re-challenge cycles with SHP-77. Error bars denote SD between donors. n = 3 (Two-way ANOVA) [file 13045_2025_1729_MOESM4_ESM.tiff]

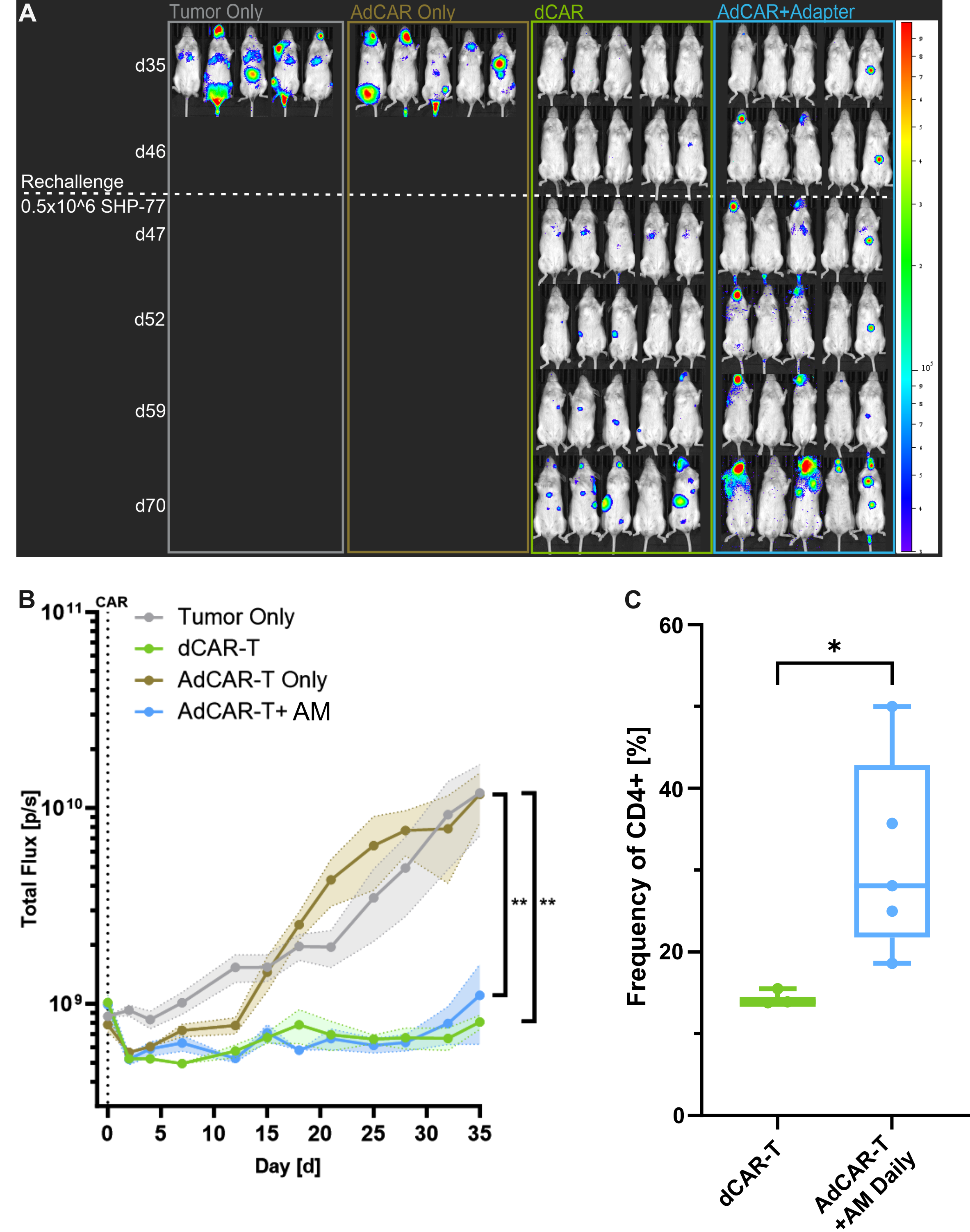

Supplement: Supplementary file 5 — Additional file 5: In vivo analysis of AcCAR-T + AM46 (A) Representative mouse images of the groups receiving no CAR-T, AdCAR-T without AM, dCAR-T or AdCAR-T with AM46. Images are on day 35 and 46 before re-challenge with 0.5x10^6^ SHP-77. Continuing with images from after re-challenge on day 47, 52, 59 and 70. n = 5. (B) Representative BLI kinetics of tumor signal of the mice with the different treatment groups on the respective imaging days. AM dosing breaks occurred between day 35 and 46. Re-challenge of the mice with 0.5x10^6^ SHP-77 occurred on day 46. Color scale represents radiance from 3x10^4^ to 1x10^6^ photons/sec. SD between mice is indicated by error bars. n = 5. **p < 0.01 (Mixed-Effect Model). (C) Analysis of the fraction of hCD45+/CD4+ cells of the treatment groups dCAR-T and AdCAR-T + AM daily on day 3 after in vivo injection into the mouse model. n = 3 for dCAR-T. n = 5 for AdCAR-T + AM Daily. *p < 0.05 (Unpaired t-test) [file 13045_2025_1729_MOESM5_ESM.tiff]
