# Supplementary material for: Targeting CD276 with Adapter-CAR T-cells provides a novel therapeutic strategy in small cell lung cancer and prevents CD276-dependent fratricide
Source: J Hematol Oncol. 2025 Jul 28;18:76. doi: 10.1186/s13045-025-01729-8 (PMC12305915; doi:10.1186/s13045-025-01729-8)
Supplement: Supplementary file 6 — Additional Table 1: Comprehensive list of the used antibodies with their respective fluorochrome, manufacturer, clone, dilution and identifier [file 13045_2025_1729_MOESM6_ESM.docx]

**Additional Table 1:** Comprehensive list of the used antibodies with their respective fluorochrome, manufacturer, clone, dilution and identifier.

| **Antibody** | **Fluorochrome** | **Manufacturer** | **Clone** | **Dilution** | **Identifier** |
| --- | --- | --- | --- | --- | --- |
| HIS | PE | Miltenyi Biotec | GG11-8F3.5.1 | 1:25 | 130-120-718 |
| Anti-IgG | FITC | Merck | CH210722.14 | 1:400 | F5512-1ML |
| Anti-Biotin | PE | Miltenyi Biotec | REA746 | 1:25 | 130-110-951 |
| CD20 | PE | Miltenyi Biotec | REA780 | 1:50 | 130-111-338 |
| CD25 | PE-Vio-770 | Miltenyi Biotec | REA945 | 1:50 | 130-116-205 |
| CD223 | BV786 | BD | T47-530 | 1:50 | 744727 |
| CD276 | - | Medac Diagnostika | RBT B7H3 | 1:600 | BSB 2813 |
| CD276 | FITC | Miltenyi Biotec | REA1094 | 1:50 | 130-118-569 |
| CD276 | PE | Miltenyi Biotec | REA1094 | 1:50 | 130-118-570 |
| CD276 | BUV737 | BD | 7-517 | 1:50 | 748379 |
| CD279 | BUV395 | BD | T47-530 | 1:50 | 744727 |
| CD366 | PE-Vio770 | Miltenyi Biotec | REA635 | 1:50 | 130-121-334 |
| CD4 | VioBlue | Miltenyi Biotec | VIT4 | 1:50 | 130-113-219 |
| CD45RA | APC | Miltenyi Biotec | REA 562 | 1:50 | 130-113-362 |
| CD62L | FITC | Miltenyi Biotec | DREG-56 | 1:50 | 304804 |
| CD69 | FITC | BD | FN50 | 1:50 | 555530 |
| CD8 | APC-Vio770 | Miltenyi Biotec | BW135/80 | 1:50 | 130-113-155 |
| hCD45 | BV711 | BD | HI30 | 1:50 | 304050 |
| mCD45 | BV605 | BD | 30-F11 | 1:50 | 563053 |
