# Supplementary material for: Targeting CD276 with Adapter-CAR T-cells provides a novel therapeutic strategy in small cell lung cancer and prevents CD276-dependent fratricide
Source: J Hematol Oncol. 2025 Jul 28;18:76. doi: 10.1186/s13045-025-01729-8 (PMC12305915; doi:10.1186/s13045-025-01729-8)
Supplement: Supplementary file 7 — Additional Table 2: Used reagents with their respective manufacturer, dilution and identifier [file 13045_2025_1729_MOESM7_ESM.docx]

**Additional Table** **2**: Used reagents with their respective manufacturer, dilution and identifier.

| **Reagents** | **Manufacturer** | **Dilution** | **Identifier** |
| --- | --- | --- | --- |
| Recombinant CD276-protein | Abcam | 1:600 | ab214154 |
| AdCAR detection reagent | Miltenyi Biotec | 1:25 | - |
